# Lysosomal lipid alterations caused by glucocerebrosidase deficiency promote lysosomal dysfunction, chaperone-mediated-autophagy deficiency, and alpha-synuclein pathology

**DOI:** 10.1038/s41531-022-00397-6

**Published:** 2022-10-06

**Authors:** Alba Navarro-Romero, Irene Fernandez-Gonzalez, Jordi Riera, Marta Montpeyo, Merce Albert-Bayo, Tresa Lopez-Royo, Pablo Castillo-Sanchez, Clara Carnicer-Caceres, Jose Antonio Arranz-Amo, Laura Castillo-Ribelles, Eddie Pradas, Josefina Casas, Miquel Vila, Marta Martinez-Vicente

**Affiliations:** 1 Neurodegenerative Diseases Research Group, Vall d’Hebron Research Institute (VHIR)-Center for Networked Biomedical Research on Neurodegenerative Diseases (CIBERNED), Barcelona, Spain; 2 Catalan Institution for Research and Advanced Studies (ICREA), Barcelona, Spain; 3 Biochemistry Service, Vall d’Hebron Hospital, Barcelona, Spain; 4 Institut de Química Avançada de Catalunya (IQAC-CSIC), Barcelona, Spain; 5 Department of Biochemistry and Molecular Biology, Autonomous University of Barcelona (UAB), Barcelona, Spain; 6 Present Address: Institut de Neurociènces and Facultad de Medicina, Autonomous University of Barcelona, Barcelona, Spain; 7 Present Address: Laboratory of Neuro-Immuno-Gastroenterology, Digestive System Research Unit, Vall d’Hebron Institut de Recerca, Barcelona, Spain; 8 Present Address: Department of Anatomy, Embryology and Animal Genetics, University of Zaragoza and CIBERNED, Zaragoza, Spain

**Keywords:** Neurodegeneration, Parkinson's disease, Cell biology, Pathogenesis

## Abstract

Mutations in the *GBA* gene that encodes the lysosomal enzyme β-glucocerebrosidase (GCase) are a major genetic risk factor for Parkinson’s disease (PD). In this study, we generated a set of differentiated and stable human dopaminergic cell lines that express the two most prevalent *GBA* mutations as well as *GBA* knockout cell lines as a in vitro disease modeling system to study the relationship between mutant *GBA* and the abnormal accumulation of α-synuclein. We performed a deep analysis of the consequences triggered by the presence of mutant GBA protein and the loss of GCase activity in different cellular compartments, focusing primarily on the lysosomal compartment, and analyzed in detail the lysosomal activity, composition, and integrity. The loss of GCase activity generates extensive lysosomal dysfunction, promoting the loss of activity of other lysosomal enzymes, affecting lysosomal membrane stability, promoting intralysosomal pH changes, and favoring the intralysosomal accumulation of sphingolipids and cholesterol. These local events, occurring only at a subcellular level, lead to an impairment of autophagy pathways, particularly chaperone-mediated autophagy, the main α-synuclein degradative pathway. The findings of this study highlighted the role of lysosomal function and lipid metabolism in PD and allowed us to describe a molecular mechanism to understand how mutations in *GBA* can contribute to an abnormal accumulation of different α-synuclein neurotoxic species in PD pathology.

## Introduction

Parkinson’s disease (PD) is an age-related neurodegenerative disorder characterized by a progressive loss of dopaminergic neurons in the *substantia nigra pars compacta* (SNpc), causing striatal dopamine depletion and, as a consequence, a combination of motor symptoms, including resting tremors, bradykinesia, and rigidity^1^. The main neuropathological hallmark of PD is the presence of protein inclusions, known as Lewy bodies, composed mainly of aggregated α-synuclein (α-syn) in surviving neurons. Several lines of evidence indicate that aberrant α-synuclein misfolding, accumulation, and aggregation are the major pathogenic processes in PD^2^. The etiology of the disorder involves an interplay of different factors, including aging, genetic susceptibility, and environmental factors. Among these factors, mutations in the *GBA* gene are the main genetic risk factor for developing PD^3,4^. *GBA* encodes the lysosomal enzyme β-glucocerebrosidase (GCase), which hydrolyses glucosylceramide (GlcCer) to ceramide and glucose. Biallelic mutations in the *GBA* gene cause Gaucher’s disease (GD), a lysosomal storage disorder characterized by decreased GCase activity and subsequent accumulation of GlcCer and glucosylsphingosine (GlcSph) in several organs. Patients with GD and mutant *GBA* carriers have a fivefold greater lifetime risk of developing PD than that in the general population^4^. L444P (c.1448T>C; p. L483P) and N370S (c.1226A>G; p. N409S) are the most common *GBA* mutations and account for 70% of all GBA mutant alleles in PD patients worldwide^5^. Both mutations result in misfolded proteins and a significant reduction in GCase activity. However, the mechanisms by which *GBA* mutations contribute to the pathogenesis of PD are not completely understood, and many different and non-exclusive hypotheses have been proposed to explain the observed relationship between alterations in GCase and α-syn pathology^6^. The loss-of-function hypothesis suggests that a decrease in GCase activity leads to substrate accumulation, which produces changes in glycosphingolipid homeostasis that, through different cellular mechanisms, can ultimately affect α-syn trafficking, processing, and clearance^7^. The gain-of-function hypothesis suggests that misfolded mutant GBA proteins are retained in the endoplasmic reticulum (ER), promoting ER stress^8^ and α-syn accumulation^9^. In addition, accumulated α-syn may impair the trafficking of GBA protein from the ER to lysosomes, resulting in a positive feedback loop that propagates disease pathology^10^. Understanding the numerous and converging underlying mechanisms leading to α-syn accumulation in PD associated with *GBA* mutations, warrant further investigation.

## Results

### Generation of an in vitro model of PD associated with GBA

**Fig. 1:**
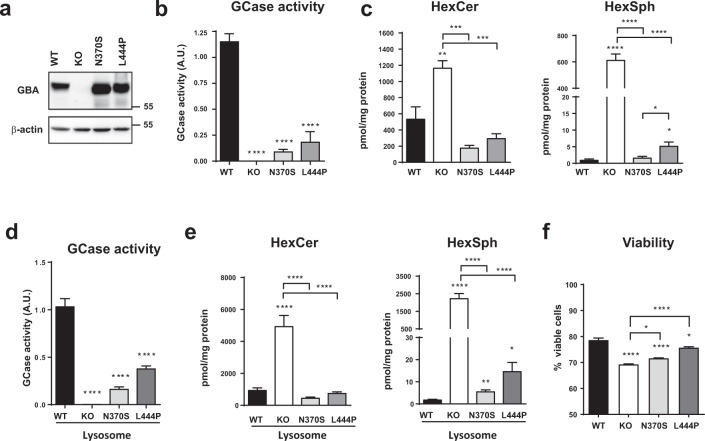
Loss of lysosomal GCase activity leads to substrate accumulation in GBA mutant cells. **a** Immunodetection of GBA protein; **b** GCase activity in total homogenate corrected by levels GBA protein, *n* = 3 in each group. **c** Quantification of total HexCer (left) and total HexSph (right) by LC/MS-MS, *n* = 3 in each group. **d** Lysosomal GCase activity in isolated lysosomal fractions, *n* = 4 independent isolations in each group. **e** Quantification of HexCer (left) and HexSph (right) by LC/MS-MS, *n* = 3–4 in each group. **f** Cellular viability, *n* = 6 independent samples. In all panels, data is presented as mean ± s.e.m. Statistical significance was established at *p < 0.05, **p < 0.01, ***p < 0.001, ****p < 0.0001 compared to WT samples or between mutant lines when indicated after one-way ANOVA followed by Tukey’s multiple comparisons test.

In the present study, we aimed to elucidate whether the lack of *GBA* or the presence of mutant *GBA* triggers lysosomal dysfunction and affects ER stress, synuclein accumulation, and cellular viability. To this end, we used differentiated human dopaminergic-like neuroblastoma BE(2)-M17 cell lines with *GBA* knockout and two of the most clinically relevant genetic variants associated with PD. First, the GBA knockout (KO) cell line was generated through the CRISPR/Cas9 system, then through stable transfection, we introduced human GBA constructs harboring the two most prevalent mutations, N370S and L444P, into this KO cell line. Expression of GBA was confirmed using western blot analysis, and after different clones were fully characterized, a representative clone of each cell line was selected (Fig. 1a). All four cell lines (GBA WT, GBA KO, GBA N370S, and GBA L444P) were characterized under differentiation conditions to study the consequences of loss of function and gain of toxic function of GBA. As expected, the GBA KO line presented minimal activity, while GBA N370S line presented 7.66% activity and GBA L444P line 15.85 % activity (related to WT GCase activity as 100%) (Fig. 1b). The conditions used to assess GCase activity exclusively ensure the detection of the activity of GBA protein (as the product of *GBA1* gene) but not cytosolic GBA2 protein^11^ (Supplementary Fig. S1a). The decrease in GCase activity resulted in an increase in the levels of GCase substrates determined using LC/MS-MS as monohexosylceramide (HexCer) and hexosylsphingosine (HexSph) species. The differentiated GBA KO cell line showed a dramatic accumulation of total HexCer and HexSph. In contrast, GBA N370S and GBA L444P presented similar levels of total sphingolipids compare to WT, with the exception of GBA L444P cells that presented a discreet increase only in HexSph levels (5.5-fold) (Fig. 1c). Overall, total sphingolipids quantification suggests that the partial GCase activity in both GBA mutant cell lines is enough to avoid the massive accumulation observed in GBA KO cells.

To further analyze the effect of GCase activity loss, we analyzed the effect of decreased lysosomal GCase activity (Fig. 1d) by analyzing pure lysosomal fractions isolated from all cell lines and validated using lysosomal markers (Supplementary Fig. S1b). The effect on sphingolipid accumulation was intensified in the lysosomal compartment, the enrichment of sphingolipids was higher in this subcellular fraction and more significant increase in the levels of HexSph substrate was observed in all mutant differentiated cell lines (Fig. 1e). The decrease in GCase activity and increase in substrate accumulation is associated with a decrease in the cellular viability of all three mutant cell lines (Fig. 1f).

### Retention of mutant GBA proteins in the ER

Lysosomal enzymes follow a specific pathway to reach lysosomes; from the ER, where they are synthesized, they travel through the Golgi apparatus, where they undergo processing, and then reach their final destination in the lysosomes. Several studies have indicated that mutant forms of GBA, including N370S and L444P, are retained in the ER^8^.

**Fig. 2:**
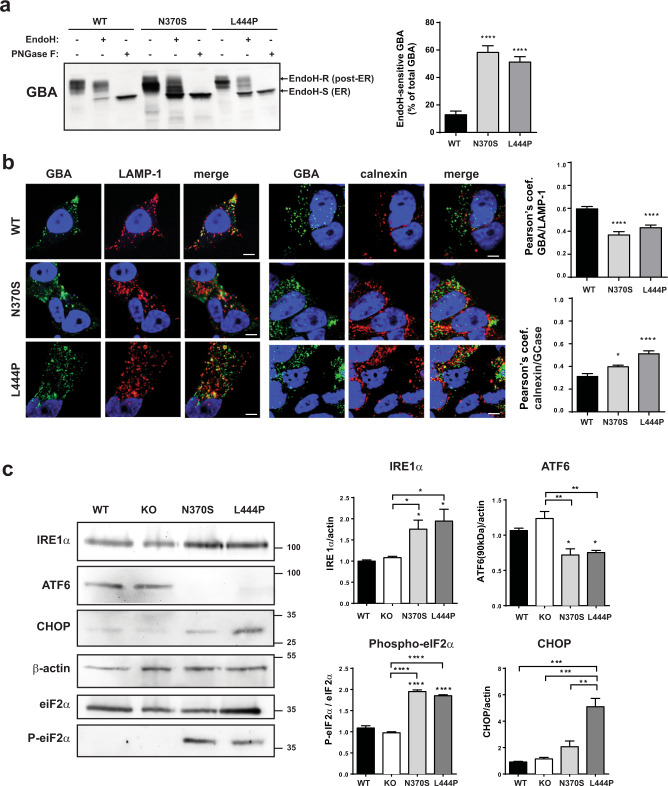
Mutant GBA proteins are retained in the ER. **a** Left: representative immunoblots of GBA before and after treatment with EndoH and PNGase F (as a positive control). **b** Right: quantification of GBA EndoH-sensitive (EndoH-S) percentage of total GBA protein, *n* = 8 independent experiments. Representative images and quantification of the colocalization of GBA with LAMP-1 (lysosomes) and calnexin (ER) determined by immunofluorescence. Colocalization was represented as Pearson’s coefficient (fraction of LAMP-1 or calnexin overlapping GBA), scale bar = 5 µM, *n* > 20 cells/condition. **c** Representative immunoblots and quantification of ER stress markers (IRE1α, ATF6, phosphorylated eIF2a, and CHOP), *n* = 3 indepe*n*dent experiments. In all panels data are presented as mean ± s.e.m, statistical significance was established at *p < 0.05, **p < 0.01, ***p < 0.001, ****p < 0.0001 compared to WT samples or between mutant lines when indicated after one-way ANOVA followed by Tukey’s multiple comparisons test.

We monitored the intracellular fate of GCase by assessing the processing of its N-glycans. Cell lysates were digested by Endoglycosidase-H (Endo-H), which specifically digests high mannose N-glycans added at the ER but not complex N-glycans present in proteins that have passed the mid-Golgi^12^. GBA L444P and GBA N370S cells were more sensitive than GBA WT cells to Endo-H restriction, showing a higher percentage of GBA EndoH-sensitive fraction, indicating that more GBA protein is retained in the ER in cell lines expressing mutant GBA proteins (Fig. 2a). No differences in the digestion were observed between groups after cells were treated with PNGase F glycosidase, which digests all sugar types. To confirm these results, we assessed the colocalization of GBA with calnexin, an ER marker, and LAMP-1, a lysosomal marker, using immunofluorescence. A lower fraction of mutant GBA than WT GBA colocalized with LAMP-1, whereas a higher fraction colocalized with calnexin (Fig. 2b). Together, these results suggest that trafficking of GBA L444P and GBA N370S from the ER to lysosomes is impaired. Accumulation of mutant GCase in the ER can lead to ER stress and to the activation of the unfolded protein response (UPR)^13^. We analyzed the levels of different ER stress markers in differentiated mutant GBA cells; mutant N370S and L444P cells but not GBA KO cells showed activation of the three branches of the UPR system^14^, as indicated by upregulation of IRE1α expression, cleavage of ATF6, and PERK-dependent hyperphosphorylation of eIF2α, ultimately leading to overexpression of the ER chaperone CHOP (Fig. 2c).

### Increase in α-synuclein species in GBA mutant cell lines

**Fig. 3:**
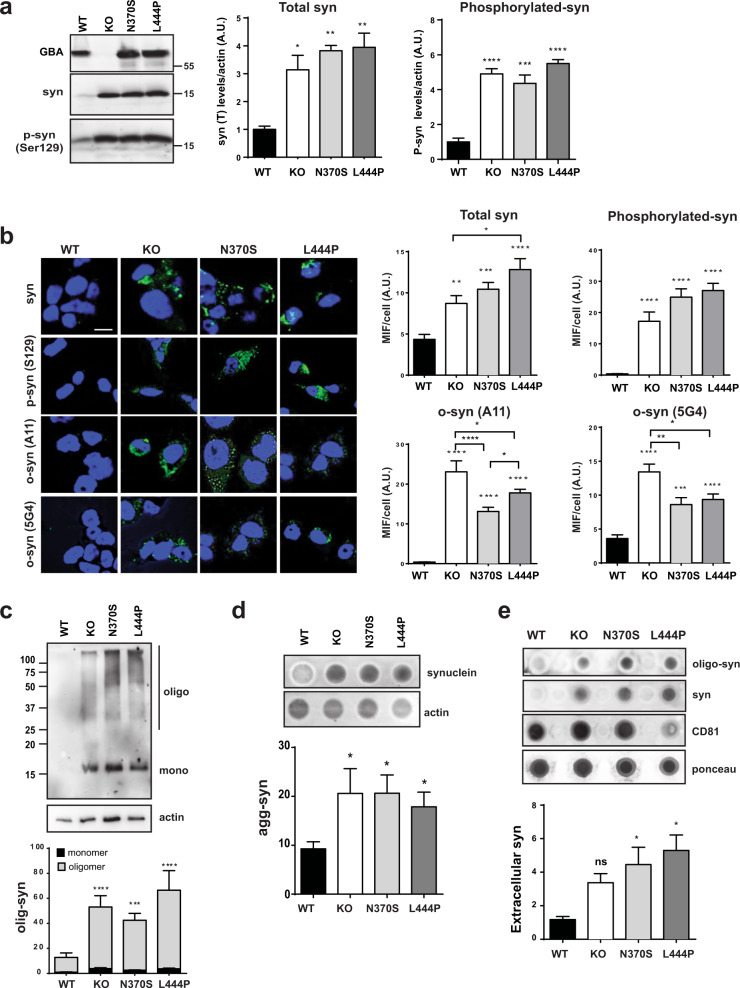
Mutant GBA accumulates different α-syn species. **a** Representative immunoblots and quantification of total synuclein and phosphorylated synuclein (Ser129). **b** Representative confocal images and quantification of the mean fluorescence intensity (MFI) of total α-syn, phosphorylated (Ser129) α-syn, and oligomeric α-syn (A11 and 5G4 antibodies) determined by immunofluorescence, scale bar = 10 µM, *n* > 20 cells/condition. **c** Immunodetection and quantification of oligomeric α-syn by in vivo cross-linking with DSG. **d** Insoluble α-syn detection and quantification using the filter retardation assay in acetate cellulose membrane after in vivo cross-linking treatment. **e** Extracellular α-syn detected by dot blotting from an extracellular culture medium with anti-synuclein and anti-oligomeric synuclein (A11); CD81 (extracellular vesicle surface protein) was used as a marker of extracellular vesicle present in the extracellular fraction. C–E: results of at least three independent experiments. In all panels, data is presented as mean ± s.e.m, statistical significance was established at *p < 0.05, **p < 0.01, ***p < 0.001, ****p < 0.0001 compared to WT samples or between mutant lines when indicated after one-way ANOVA followed by Tukey’s multiple comparisons test.

Several studies on PD models and patient samples have revealed an association between GCase activity loss and α-syn accumulation^6,10^. We measured different intracellular α-syn species using immunodetection; in GBA KO, L444P, and N370S cells, total and phosphorylated α-syn (Ser129) levels were increased in comparison with those in WT cells (Fig. 3a). In addition, other neurotoxic forms of α-syn, such as oligomeric α-syn species, were quantified using the specific high molecular weight β-sheet oligomer A11 and 5G4 antibodies. All mutant cell lines displayed increased α-syn oligomer signals compared with those of WT cells, confirming that loss of GCase protein and all mutant forms of GCase promote a clear increase in total α-syn levels but, more importantly, a drastic increase in the levels of neurotoxic forms of α-syn (Fig. 3b).

To further study the presence of α-syn oligomers, we analyzed the levels of these α-syn species in cell lysates using the chemical cross-linker disuccinimidyl glutarate (DSG), which forms irreversible amide bonds between interacting proteins. Immunoblotting revealed that the levels of α-syn oligomeric species were significantly increased in GBA KO and mutant cells (Fig. 3c). We also analyzed the levels of insoluble fibrillary α-synuclein aggregates using a filter retardation assay^15^. Results indicated that mutant cells presented increased levels of insoluble α-synuclein aggregates retained in the acetate cellulose filter (Fig. 3d).

Analysis of the culture media of differentiated BE(2)-M17 cells revealed increased levels of extracellular monomeric and β-sheet-rich oligomeric forms of α-syn in GBA KO, N370S, and L444P cells (Fig. 3e).

### Lysosomal dysfunction in GBA mutant cell lines

Lysosomal dysfunction has been described as one of the main mechanisms involved in PD etiology^16^, mainly owing to the consequences of deficits in lysosomal-dependent α-syn turnover that leads to α-syn accumulation. Soluble α-syn is predominantly degraded by lysosomal pathways, particularly CMA; however, oligomeric, aggregated, and some modified α-syn species cannot be eliminated by this pathway and are degraded by macroautophagy^17^. Failure in lysosomal activity can affect both macroautophagy and CMA pathways and promote α-syn accumulation, which can trigger pathogenic α-syn aggregation. To further investigate the connection between GBA and α-syn, we analyzed lysosomal activity and autophagy pathways in detail using different methodologies.

**Fig. 4:**
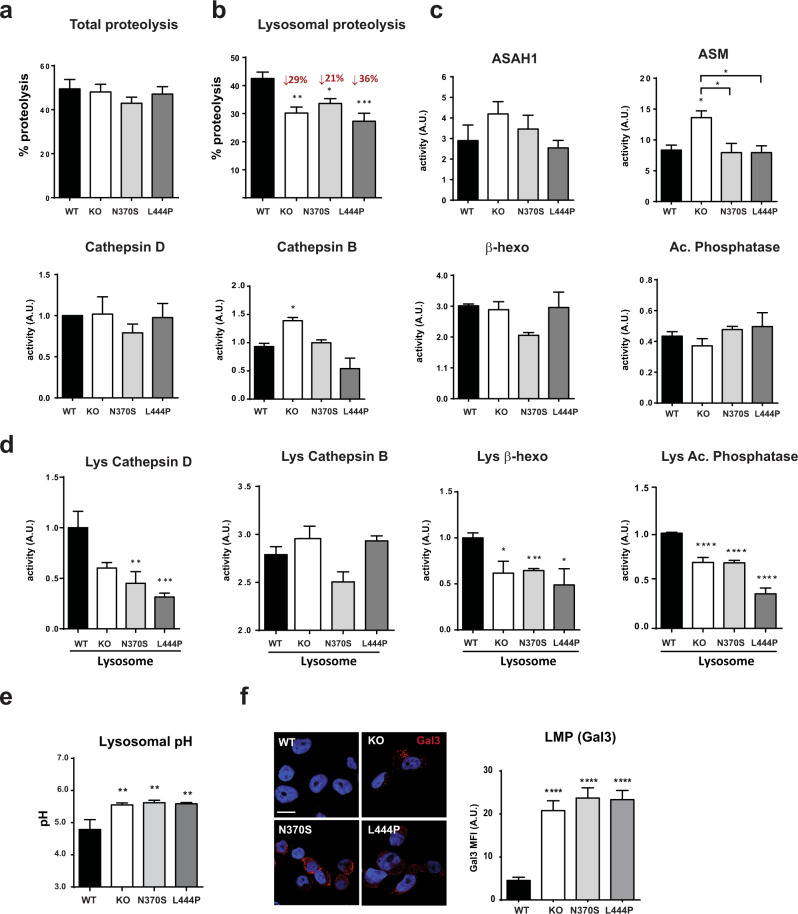
Mutant GBA exhibit lysosomal dysfunction. **a** Total proteolysis measured by an intracellular protein assay, *n* = 12. **b** Lysosomal proteolysis measured by an intracellular protein assay, *n* = 12. **c** Enzymatic activity of lysosomal enzymes in total homogenates, *n* = 3–6 independent samples. **d** Enzymatic activity of lysosomal enzymes in lysosomal-enriched fractions, *n* > 3 independent isolations in each group. **e** Lysosomal pH quantification, *n* = 6 in each group. **f** Representative images and quantification of galectin-3 (red) determined by immunofluorescence. Nuclei are labeled with Hoechst 33342, scale bar = 10 µm, *n* = 30 cells/conditions. In all panels, data are presented as mean ± s.e.m. Statistical significance was established at *p < 0.05, ** *p* < 0.01, ***p < 0.001, ****p < 0.0001 compared to WT samples or between mutant lines when indicated after one-way ANOVA followed by Tukey’s multiple comparisons test.

First, we performed a live-cell proteolysis assay to quantify total proteolysis (Fig. 4a) and lysosomal-dependent proteolysis (Fig. 4b) by measuring the degradation rate of long-lived proteins^18^. No differences were observed in total proteolysis, indicating that the lack of GBA or the presence of mutant forms of GBA under basal conditions does not directly affect the efficiency of total cellular degradation. However, when analyzing only lysosomal degradation, which was measured as proteolysis sensitive to ammonium chloride and leupeptin, a clear decrease in the lysosomal-dependent proteolysis rate was observed.

To further investigate lysosomal dysfunction, we analyzed the enzymatic activity of a series of representative lysosomal enzymes, including cathepsins D and B, β-hexosaminidase, acid phosphatase, N-acylsphingosine amidohydrolase (ASAH1), and acid sphingomyelinase (ASM). Overall, no remarkable changes were observed in the activity of these lysosomal enzymes in total lysates from the different cell lines, with some exceptions; ASM and cathepsin B activities were increased only in GBA KO cells (Fig. 4c). However, when we focused on the lysosomal compartment and analyzed enzymatic activity in the lysosome-enriched fraction instead of the total homogenate, we observed a significant decrease in the activity of all lysosomal enzymes in all three mutant cell lines except cathepsin B (Fig. 4d), demonstrating the importance of analyzing the enzymatic activity of lysosomal enzymes in isolated lysosomes. This decrease was also confirmed by studying the live-cell enzymatic activity of cathepsin D in vivo using BODIPY-FL-pepstatin A and quantifying the levels of mature cathepsin D and B present in the lysosomal fraction (Supplementary Fig. S2), corroborating that GCase activity loss is associated with a decrease in the enzymatic activity of several other representative lysosomal enzymes.

We also analyzed other lysosomal features such as lysosomal pH and membrane permeabilization that can be altered because of the accumulative events triggered by the loss of lysosomal GCase. We analyzed the lysosomal pH in all cell lines using LysoSensor™ Yellow/Blue DND-160 and observed that all mutant cell lines showed an increased lysosomal pH, which can contribute to deficient maturation of lysosomal enzymes and reduced degradation rates (Fig. 4e). In addition, the integrity of the lysosomal membrane was monitored by quantifying the protein level of galectin-3, a marker of lysosomal membrane damage^19^. Using immunofluorescence analysis performed in differentiated KO and mutant GBA, a clear increase in the number of galectin-3 puncta patterns by neurons was observed (Fig. 4f).

### GBA mutant cell lines present a slight effect on macroautophagy

**Fig. 5:**
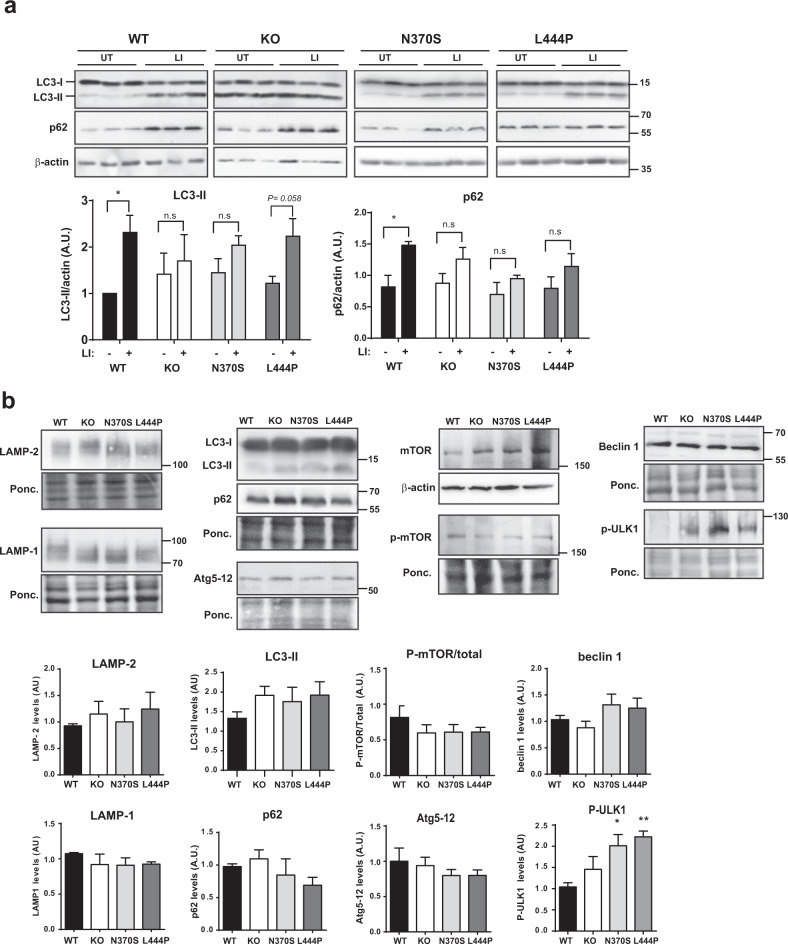
Mutant GBA causes alterations in macroautophagy. **a** Autophagic flux: immunodetection and quantification of LC3-II and p62 in the presence and absence of the lysosomal inhibitor (LI) 60 µM chloroquine. Results are presented as mean ± s.e.m. values of three independent experiments. Statistical significance was established at *p < 0.05 compared to untreated conditions by two-tailed unpaired Student’s *t*-test. **b** Representative immunoblots and quantification of autophagic and lysosomal markers. In all panels, results are presented as mean ± s.e.m. values of at least six independent experiments. Statistical significance was established at *p < 0.05, **p < 0.01, compared to WT samples after one-way ANOVA followed by Tukey’s multiple comparisons test.

As a consequence of the lysosomal dysfunction, the autophagy process might be affected; accordingly, we analyzed both macroautophagy and CMA. Macroautophagy activity has been studied in several GBA models with different and contradictory results^20–22^. The analysis of autophagic flux showed that in WT cells the addition of the lysosomal inhibitor can significantly increase LC3-II levels, however in the mutant cells including KO, although there is a slight increase of LC3-II after the inhibition, this increase is not significant, indicating that there is a deficiency in autophagy flux (Fig. 5a). This partial impairment of autophagic flux under basal conditions was also assessed by quantifying the levels of p62/SQSTM1, revealing that the degradation of autophagosomes within lysosomes was partially compromised. We further analyzed the levels of some key macroautophagy markers in all mutant differentiated cell lines (Fig. 5b). Overall, we did not observe significant changes in the levels of these markers except for phosphorylated ULK1 expression, which was significantly increased in GBA mutant cells, suggesting a possible compensatory promotion of macroautophagy at the initiation step (Fig. 5b). A tendency for beclin 1 levels to be increased and phosphorylated mTOR levels to be decreased could support this hypothesis. No significant changes were observed in the other macroautophagy markers confirming that reasonably regular macroautophagy activity is maintained under basal conditions.

### GBA deficiency leads to lysosomal lipid modifications that affect CMA activity

**Fig. 6:**
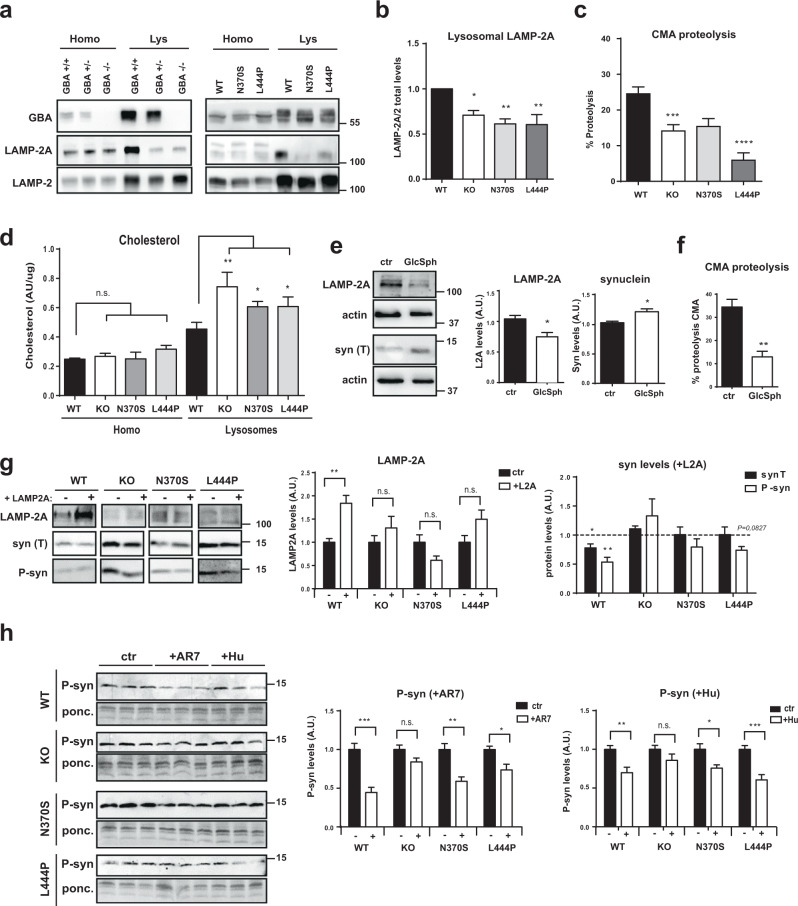
Mutant GBA is associated with CMA impairment. **a** Immunodetection of LAMP-2A, total LAMP-2, and GBA in total homogenate (homo) and isolated lysosomes (Lys). **b** Quantification of LAMP-2A levels in the lysosomal fraction corrected using total LAMP-2, *n* = 4 independent isolations. **c** Proteolysis dependent on CMA detected as 3-MA-insensitive/leupeptin-NH_4_Cl-sensitive proteolytic activity, *n* > 4 in each group. **d** Total cholesterol quantification in homogenate (homo) and isolated lysosomes (Lysosomes) *n* = 6. **e** Quantification of total LAMP-2A and total synuclein levels in GBA WT cell lines after 72 h treatment with 5 mM GlcSph, *n* = 3. **f** Proteolysis dependent on CMA detected as 3-MA-insensitive/leupeptin/NH_4_Cl-sensitive proteolytic activity after 72 h treatment with 5 mM GlcSph, *n* = 3. **g** Representative images (left) of immunodetection of LAMP-2A and total/phosphorylated synuclein after 48 h of transient transfection. Quantification (right) of LAMP-2A transfection (LAMP-2A levels in control transfection in each cell line normalized to 1) and total and phosphorylated α-syn after transfection (syn protein levels in control transfection in each cell line normalized to 1), values of three independent experiments. **h** Immunodetection of phosphorylated synuclein (P-syn) and ponceau (ponc) as a loading control after 24 h treatment with AR7 and humanin (Hu) (left), and quantification of P-syn levels (left) [P-syn levels in cells treated with vehicle (ctr) in each cell line are normalized to 1]; values of six independent samples. In all panels, results are presented as mean ± s.e.m values. Statistical significance was established at * *p* < 0.05, **p < 0.01 after two-way ANOVA followed by Tukey’s multiple comparisons tests (**b**–**d**) or two-tailed unpaired Student’s *t*-test (**e**–**h**).

We next questioned whether lysosomal dysfunction could affect CMA, especially considering the important role of this autophagic pathway in α-syn degradation and PD pathophysiology and that lower levels of LAMP-2A and CMA activity have been reported in PD models and samples from PD patients^23–25^. We first quantified the levels of the LAMP-2A isoform (Fig. 6a) since CMA activity depends on the lysosomal receptor complex formed by the oligomerization of the lysosomal protein LAMP-2A^26^. Interestingly, total LAMP-2A level was not changed in mutant or KO GBA cells. However, when analyzing the LAMP-2A levels exclusively in the lysosomal fraction, LAMP-2A levels were drastically decreased when GBA was knocked down or mutated. As expected, this decrease in lysosomal LAMP-2A expression (Fig. 6b) correlated with a decrease in CMA activity quantified by the proteolysis activity assay as ammonium chloride and leupeptin-sensitive and 3-MA-insensitive proteolysis^27^ (Fig. 6c).

The stability and activity of LAMP-2A are determined in part by the lipid microdomains located in the lysosomal membrane that are mostly enriched in sphingolipids and cholesterol. We first analyzed the transcriptional expression of the LAMP-2A isoform in the different cell lines (Supplementary Fig. S3) showing no significant changes in the expression of LAMP-2A and suggesting that decreases in LAMP-2A might be a consequence of its increased turnover inside the lipid microdomains^28,29^. In addition to the high levels of GlcSph previously observed in lysosomal fractions (Fig. 1e), we also analyzed cholesterol levels and observed an increase in mutant GBA cell lines in the lysosomal-enriched fraction (Fig. 6d). Similarly, when the sphingolipid GlcSph was added to WT GBA cell lines, we observed decreased LAMP-2A levels (Fig. 6e) and increased α-syn levels associated with decreased CMA activity (Fig. 6f).

To confirm that the loss of function of GBA was responsible, at least in part, for the increase in α-syn levels through alterations in lysosomal LAMP-2A levels and CMA activity, we attempted to restore LAMP-2A levels in mutant cell lines through the expression of the LAMP-2A isoform by transient transfection. Interestingly, the attempt to overexpress LAMP-2A in the GBA WT cell line was associated with a significant decrease in P-syn and total α-syn levels (Fig. 6g). However, in the KO and mutant cell lines, transfection of LAMP-2A protein was not efficient, probably because high sphingolipid and cholesterol levels in these cell lines prevent the stabilization of LAMP-2A levels in the lysosomal membrane and proper assembly of the CMA-translocation complex. Accordingly, P-syn and total α-syn levels were not significantly decreased in these cell lines (Fig. 6g).

Alternatively, pharmacological activation of CMA without directly targeting LAMP-2A protein with AR7 and humanin treatment^30,31^ was more efficient in promoting phosphorylated synuclein clearance in WT, N370S, and L444P cell lines, but not in KO cells where accumulated P-syn was not significantly decreased (Fig. 6h).

## Discussion

In recent years, numerous non-exclusive mechanisms have been proposed to participate in the link between GBA and PD (reviewed in^10^). In our studies, we used differentiated BE(2)-M17 immortalized cell lines of human neuronal origin, presenting a well-characterized dopaminergic phenotype, neurite extension, and arrest of cellular proliferation^32^. These cells were generated and analyzed in parallel; thus, GBA KO was used as a model of complete loss-of-function and maximal accumulation of substrates, and mutant GBA cell lines (L444P and N370S) as models of gain-of-toxic function owing to the presence of aberrant mutant proteins as well as partial loss of enzymatic activity (Fig. 1).

The link between GCase activity and α-syn may explain the relationship between GBA mutations and PD. We could observe that the loss of GCase activity and the presence of mutant forms of the GBA protein was clearly associated with an increase in different neurotoxic forms of α-syn associated with PD, including oligomeric, insoluble, phosphorylated and extracellular species (Fig. 3a–e); the aim of this work was to identify underlying pathways leading to α-syn accumulation in our GBA in vitro model.

As expected, the GBA KO cell line accumulates GlcCer. However, it more dramatically accumulates GlcSph, the main sphingolipid accumulated in GBA deficient cells and used as Gaucher’s diagnosis biomarker^33^, while N370S and L444P cell lines maintain a partial GCase activity that avoids this massive sphingolipid accumulation. Previous studies analyzing sphingolipid levels in GBA-related models showed variable results, with a clear accumulation of sphingolipids in homozygous GBA mutant models^34–38^ and not so clear accumulation in heterozygous GBA mutant models^38,39^. In human studies, some authors reported elevated levels of both substrates in the brains of neuropathic GD patients^40,41^ and significantly increased levels in the substantia nigra of sporadic PD brains^42,43^. In contrast, other studies reported no changes in GlcCer and GlcSph levels in different brain areas of PD patients with GBA variants^44–46^. This lack of a clear data on accumulation of GBA substrates in some PD-GBA models and samples from PD patients carrying heterozygous GBA mutations might suggest that residual GCase activity may be enough to avoid the massive accumulation of GlcCer and GlcSph and raises concerns about the role of these sphingolipids in the pathogenesis of PD. However, this apparent discrepancy might be explained by the types of samples analyzed in previous studies. Based on the results of our study, many of the lysosomal-related deficits revealed in our model were only noticeable when lysosomal-enriched samples instead of total homogenates were used. Thus, the quantification of lipids in the lysosomal fraction revealed significantly increased levels of GlcSph and cholesterol (Figs. 1e and 6d) that were not detectable in the total homogenate of the same samples (Figs. 1c and 6d), suggesting that accumulation of sphingolipids, cholesterol, and other related lipids can occur and are detectable only locally. Additionally, as an aggravating effect, these accumulated lipids can also be transported to other membranes in other subcellular localizations such as mitochondria^22^ and the ER^47^. Moreover, since they are bioactive lipids and components of several signaling networks, they can trigger other pathogenic effects. Thus, in addition to the consequences of lysosomal accumulation, we cannot ignore the fact that increased levels of GlcCer, GlcSph, and other lipids such as gangliosides and GlcChol produced because of GCase impairment might alter cellular homeostasis and facilitate neuronal cell death through different mechanisms beyond the lysosome^48–50^.

The gain-of-toxic-function hypothesis mainly focuses on the consequences of the accumulation of mutant GBA proteins in the ER. It has been extensively reported that in fibroblasts and iPSC-derived dopaminergic neurons from PD patients carrying GBA mutations, mutant GBA is retained in the ER^8^. In our neuronal model, we clearly confirmed the retention of mutant GCase in the ER (Fig. 2a, b) and the subsequent activation of the UPR (Fig. 2c) in differentiated L444P and N370S mutant cell lines. We hypothesize that the chronic ER stress generated by the sustained accumulation of mutant GBA proteins in the ER probably triggers other interconnected pathways with synergistic effects, including autophagy alterations, oxidative stress, and mitochondrial dysfunction^51^. In addition, a new gain-of-toxic-function mechanism beyond the ER has been recently reported, where a fraction of mutant GBA that fails to fold in the ER is targeted to lysosomes to be eliminated by CMA; however, mutant GBA is retained in the lysosomal membrane and blocks the multimerization of LAMP-2A, impairing the normal uptake and degradation of other CMA substrates such as alpha-synuclein^52^.

After validating the in vitro model, the main aim of this work was to analyze in detail the role of lysosomal-related mechanisms in PD-associated GBA. Lysosomal dysfunction has long been described as one of the main mechanisms involved in PD etiology^16^, mainly because of the impairment of α-syn turnover. In both non-neuronal cell lines such as patient-derived fibroblasts^21,53^ and neuronal cell lines such as dopaminergic neurons derived from iPSCs and SH-SY5Y cells^20,21,54–57^, different studies have reported varied and contradictory alterations in the activity of specific lysosomal enzymes in total lysates^33^. In our study, we measured the activity of different representative lysosomal enzymes in the lysosomal fraction as a readout of lysosomal function (Fig. 4d and Supplementary Fig. S2). We detected a clear impairment of individual enzymatic activity that was observable only in the lysosomal fraction but not in the total homogenate (Fig. 4c). The discrepancies among previous studies might be explained by the fact that measuring activities in total homogenates does not allow for discriminating the enzymatic activity of the enzymes present within the lysosome from that of those present in other subcellular compartments such as the ER or Golgi, leading to an overestimation of the enzymatic activity of lysosomal enzymes in total homogenates. Accordingly, although total proteolysis did not seem to be affected in our differentiated neuronal model (Fig. 4a), the quantification of lysosomal-dependent proteolysis showed a significant decrease under basal conditions in all mutant cells relative to that in WT cells (Fig. 4b).

Changes in the acidification of the lysosome can affect intralysosomal processing and the efficiency of lysosomal enzymes^58^. Moreover, changes in the intralysosomal pH have been associated with different pathologies, including PD^59^. In the mutant cell lines, we observed an increased intralysosomal pH and lysosomal membrane permeabilization (Fig. 4e, f) that illustrate the consequences of GCase activity loss on lysosomal function.

This lysosomal dysfunction could reciprocally affect the different forms of autophagy. Some preliminary macroautophagy studies have reported increased levels of the autophagosome marker LC3-II and the macroautophagy substrate p62/SQSTM1 under basal conditions in neuronal^22,54,55,60^ and non-neuronal^61^ in vitro models, but again other in vitro studies reported conflicting results with no changes in macroautophagy markers^33^. In the mutant GBA cell lines, we did not detect a significant increase in the levels of these two autophagy-related proteins under basal conditions (Fig. 5b). Nevertheless, the analysis of autophagic flux confirmed that although new autophagosomes were formed, fused with lysosomes, and degraded, this process was significantly diminished but probably still partially functional (Fig. 5a). Macroautophagy marker studies indicated normal levels of these proteins but with a tendency to show an activated induction of macroautophagy under basal conditions in the mutant cell lines (phosphorylated ULK1 in Fig. 5b). This suggested a compensatory response for the partial loss of lysosomal function and autophagy activity as previously found in other models^62^.

Finally, our work focused on the CMA mechanism since this autophagy pathway plays a key role in PD. CMA is the main degradative pathway by which intracellular α-syn is eliminated^17,24^. Consequently, a decrease in CMA activity has been associated with an increase in α-syn levels in PD patient samples and in vivo and in vitro models^23,63–65^. In our neuronal model, we analyzed CMA activity (Fig. 6c) and the lysosomal levels of LAMP-2A (Fig. 6a, b). Both parameters were affected in the KO and mutant cell lines. The observed decrease in CMA activity can be attributed to decreased levels of LAMP-2A; nevertheless, the previously reported dysfunction of lysosomal proteolysis and reduced enzymatic activity of several lysosomal enzymes might also have a summation effect on CMA impairment.

Interestingly, in line with previous results, total LAMP-2A expression was not significantly altered in homogenates but drastically decreased in the lysosomal fraction (Fig. 6a, b). Previous studies showed that a decrease in LAMP-2A receptor expression or blockage of LAMP-2A receptor translocation to the lysosomal membrane leads to accumulation of α-syn^65–68^. In addition, LAMP-2A levels and translocation complex assembly depend on lipid microdomains on the lysosomal membrane. Higher levels of sphingolipids, gangliosides, and cholesterol, the main components of lipid microdomains, favor an increase in the number of lipid microdomains present in the lysosomal membrane and consequently the presence of LAMP-2A as a monomer, preventing the assembly of the CMA-translocation complex and inhibiting CMA activity^69^. We confirmed that the low levels of LAMP-2A present in the lysosomal membrane might be due to post-translational mechanisms (Supplementary Fig. S3) and the levels of GlcSph, GlcCer (Fig. 1e, f), and cholesterol (Fig. 6d) were increased in the lysosomal fraction, which could explain the higher LAMP-2A turnover and consequently the decrease in CMA activity.

The correlation between the decrease in LAMP-2A levels and α-syn turnover caused by the accumulation of GBA substrates was confirmed by the addition of high levels of GlcSph to the GBA WT cell lines, which promoted a decrease in LAMP-2A expression, CMA impairment, and α-syn accumulation (Fig. 6e, f). To revert the effect of CMA impairment, genetic overexpression of the LAMP-2A isoform and the subsequent clearance of synuclein isoforms were only effective in GBA WT cell lines (Fig. 6g). In the cell lines presenting lysosomal sphingolipid and cholesterol accumulation, the genetic overexpression of the LAMP-2A isoform was probably unable to maintain stable levels of the LAMP-2A complex due to continuous LAMP-2A degradation within the lysosomal lipid rafts (Fig. 6g). Pharmacological activation of CMA without directly targeting LAMP-2A protein with AR7 and Hu (Fig. 6h) was more efficient in promoting phosphorylated synuclein clearance in WT, N370S, and L444P cell lines but not in KO cells which express higher sphingolipid and cholesterol levels in the lysosomal fraction. Overall, these experiments confirmed that abnormally high levels of sphingolipids and cholesterol in the lysosomal membrane of GCase-deficient neurons could affect CMA activity and consequently the degradation of the CMA substrate α-syn. We cannot dismiss the fact that alternative mechanisms can also directly or indirectly impair CMA activity, as previously observed, due to high levels of α-syn, which can favor CMA impairment^52,70^. Finally, we also consider that CMA-independent mechanisms based on the abnormal accumulation of sphingolipids and cholesterol can also influence α-syn localization, oligomerization, and physiological processes^71^.The direct interaction of α-syn with these lipids can promote the formation of oligomeric and toxic forms of α-syn on the lysosomal membrane^71,72^, suggesting that accumulated sphingolipids and cholesterol in lysosomes might act as aggregation sites for α-syn.

In conclusion, in this study, we defined a neuronal model that recapitulated the most important alterations in GBA-associated PD and described a new molecular mechanism to understand the etiology of GBA-associated PD (see graphical abstract, Supplementary Fig. S4). Importantly, most of the noticeable features of GBA-dependent lysosomal dysfunction are only significantly evident in the lysosomal compartment. Our results showed that the increase in lysosomal lipids associated with GCase activity loss could also lead to CMA activity impairment by destabilizing the LAMP-2A receptor at the lysosomal membrane. This impairment of CMA activity promotes the abnormal accumulation of α-syn and joins other synergistic and convergent pathways that contribute to altering α-syn levels, solubility, and conformation, ultimately promoting neurodegeneration.

## Methods

### Culture cell lines

*BE(2)-M17 cell line:* human neuroblastoma cell line BE(2)-M17 was maintained in Opti-MEM supplemented with 10% fetal bovine serum, 1% penicillin–streptomycin, and 0.5 mg/mL active geneticin, all from Thermo Fisher Scientific (Waltham, MA, USA). Cells were differentiated into dopaminergic-like neurons in Neurobasal medium supplemented with 10% inactivated fetal bovine serum, 1% penicillin–streptomycin, 0.5 mg/mL geneticin, B-27 Supplement, and 10 μM retinoic acid, all from Thermo Fisher Scientific, for at least 7 d. *Generation of GBA knockout (KO) BE(2)-M17 cells by CRISPR/Cas9 nickase system*: GBA KO cell line was created using the CRISPR/cas9 nickase methodology. sgRNAs that target exon 3 of the human GBA1 genomic sequence were subcloned into the pSpCas9n(BB)-2A-GFP (PX461) plasmid (Plasmid #48140; Addgene, Watertown, MA, USA) according to the manufacturer’s protocol. sgRNA sequences were designed using Benchling software (www.benchling.com) and consisted of two pairs of oligonucleotides: 5′-CAC CGTGTACTCTCATAGCGGCTGA-3′ and 5′-AAACTCAGCCGCTATGAGAGTACAC-3′; 5′-CACCGTACACGCAGAGGGCGACGGA-3′ and 5′-AAACTCCGTCGCCCTCTGCGTGTAC-3′. Both plasmids were validated and co-transfected into BE(2)-M17 cell lines. After 48 h, cells were sorted in a BD FACScaliburTM flow cytometer (BD Biosciences, Franklin Lakes, NJ, USA), and GFP-positive cells were re-seeded at low density and cultured until single cells formed cell colonies. Thereafter, single colonies were picked and analyzed by assessing GCase levels using immunoblot, GCase activity, and sequencing. At least five GBA KO cell lines were fully characterized to confirm the complete loss of GCase protein. *Mutant GBA plasmids, transfection, and generation of mutant GBA stable cell lines*: site-directed mutagenesis was performed into an original pCMV3-hGBA plasmid (HG12038-UT; Sino Biological, Beijing, China) to generate pCMV3-hGBA-N370S and pCMV3-hGBA-L444P plasmids using the primers pairs 5′-GCCACAGCATCATCACGAGCCTCCTGTACCATGTGG-3′ with 5′-TGTACTGCATCCCTCGATCCCAGGAGCCTAGCCGCA-3′ and 5′-GTCAGAAGAACGACCCGGACGCAGTGGCACTG-3′ with 5′-TGGCAACCAGCCCCACTCTCTGGGAGCCCTCAG G-3′, respectively, and a Q5 Site-Directed Mutagenesis Kit (New England Biolabs, Ipswich, MA, USA) following the manufacturer’s instructions. Plasmids expressing N370S GBA or L444P GBA were transfected into BE(2)-M17 GBA KO cells, and stable colonies were generated after puromycin selection. Subsequently, single colonies were selected and validated by sequencing, immunodetection, and GCase activity. At least five single colonies were fully characterized, and one clone was selected as a representative.

### Reagents and antibodies

*Reagents*: leupeptin hemisulfate salt (L2884), ammonium chloride (A9434), 3-methyladenine (3-MA, M9281), carbonyl cyanide 3-chlorophenyl hydrazone (CCCP, C2759), oligomycin (75351), antimycin A (A8674), AR7 (SML0921), chloroquine (C6628) and conduritol β epoxide (C5424) were obtained from Sigma-Aldrich (St. Louis, MO, USA). Protease/Phosphatase Inhibitor Cocktail (5872) was purchased from Cell Signaling Technology (Danvers, MA, USA), and the Protease Inhibitor cocktail (A32965) and BCA Protein Assay Kit (23225) were obtained from Thermo Fisher Scientific. d-glucosyl-β1-1′-d-erythro-sphingosine (GlcSph) (860535P) was obtained from Avanti Polar Lipids Inc (Alabaster, AL, USA) and humanin (ALX-158-011-M001) from Enzo Life Sciences Inc (Farmingdale, NY, USA). *Antibodies:* LC3 (NB-2220), ULK1 (NBP2-24738S), ATF6 (NBP1-40256S), LAMP-2 (sc-18822), LAMP-1 (sc-19992), beclin 1/BECN1 (sc-48341), CD81 (D-4) (sc-166028), Grp78/BiP (sc-376768), Atg 5 (sc-133158), and cathepsin D (sc-6494) were obtained from Santa Cruz Biotechnology (Dallas, TX, USA). GBA (ab55080) and phospho-α-syn (pS129) (ab51253) were purchased from Abcam (Cambridge, UK). Phospho-ULK1 Ser757 (6888S), eIF2α (9722), IRE1α (14C10) (3294), Phospho-eIF2α (Ser51) (9721), Phospho-mTOR (2971S), mTOR (2972S), and CHOP (2895) were obtained from Cell Signaling Technology. Cathepsin B (219408) was obtained from Calbiochem (San Diego, CA, USA), SQSTM1/p62 (GP62-C) from Abnova (Taipei, Taiwan), β-actin (A5441) from Sigma-Aldrich, Oligomer A11 (AHB0052) and Calnexin (MA3-027) from Thermo Fisher Scientific, α-syn (610786) from BD Biosciences, and aggregated α-syn (MABN389) from Merk Millipore (Burlington, MA, USA). Secondary HRP antibodies were purchased from GE Healthcare (Chicago, IL, USA), and Secondary Alexa Fluor 488 and 594 were obtained from Thermo Fisher Scientific.

### Sphingolipid quantification

Lipid extraction was performed based on the methodology described in ref. ^73^. Briefly, frozen cell pellets were dissolved in 540 µL of IS Working Solution [MeOH:Cl_3_CH (2:1) solution containing 0.01% of butylated hydroxytoluene (BHT), 1 nM internal standard glucosyl(ß)-sphingosine-d5 and 13 nM C18 glucosyl(ß)-ceramide-d5 solution (Avanti Polar Lipids Inc; 860636P, 860638P)]. Samples were vortexed, sonicated, and centrifuged at 13,000 rpm for 5 min. The supernatant was evaporated under an N_2_ stream until dry and reconstituted with 500 µL of MeOH:Cl_3_CH (1:2). Next, a seven point calibration curve (from 0.0275 to 100 pmol) was prepared by spiking a working solution containing different concentrations of GlcSph (Matreya LLC, State College, PA, USA), GlcCer C16:0, C18:0, and C24:1 (860539 P, 860547P, 860549P; Avanti Polar Lipids Inc) standards in MeOH:Cl_3_CH (1:2). Analysis of total hexosylsphingosine (HexSph) and hexosylceramide (HexCer) isoforms (C16:0, C18:0, C20:0, C22:0, C24:1, C24:0) was performed by liquid chromatography-mass spectrometry (LCMS-8050; Shimadzu, Kyoto, Japan) using an HPLC reversed-phase column (TR-10902; Teknokroma Analítica S.A., Barcelona, Spain) with a binary gradient of 0.1% formic acid in water (mobile phase A) and 0.1% formic acid in isopropanol:acetonitrile 3:1 (v/v) (mobile phase B). The mass analyzer was operated in positive ion electrospray mode. The HexSph and HexCer species were quantified using their respective standard calibration curves using LabSolutions CS Software.

### Total homogenate and subcellular fractionation from cells

Differentiated cells were harvested in PBS and pelleted by centrifugation at 800 × *g* for 5 min. Total homogenates were extracted in conventional lysis buffer (20 mM Tris pH 7.5, 1 mM EDTA, 150 mM NaCl, 1% triton and protease and phosphatase inhibitors (Cell Signaling, 5872). To isolate subcellular fractionations cellular pellets were resuspended in 0.25 M sucrose (pH 7.2) and disrupted in a cavitation chamber (Kontes Glass Company) followed by homogenization in a Teflon-glass homogenizer and centrifugation (2500 × *g* for 15 min). Supernatant was then centrifuged at 17,000 × *g*. The supernatant was collected and ultracentrifuged 1 h at 100,000 × g to collect cytosolic fraction, while the resulting pellet after the 17,000 × *g* centrifugation was collected as the lysosomal/mitochondrial-enriched fraction and was then loaded in two subsequent discontinuous Nycodenz, sucrose or percoll (GEHealthcare, 17-0891-01) density gradient from which pure lysosomal fraction was collected according to^74^. Protein concentration of all fractions was determined by the bicinchoninic assay method with BSA as a standard protein and samples were frozen and stored at −20 °C until used.

### Immunoblot detection

Cellular samples (total homogenate or isolated fractions) were analyzed by western blot, samples were resolved by SDS-PAGE on different percentages of polyacrylamide gels, ranging from 7% to 15%. Resolved proteins were transferred onto nitrocellulose membranes and blocked with 5% non-fat milk before incubation with the corresponding primary antibody (diluted in 4% bovine serum albumin in PBS) and subsequently in the corresponding secondary antibody coupled to horseradish peroxidase (diluted in 5% non-fat milk in PBS). Proteins were visualized using either West Pico SuperSignal Substrate or SuperSignal West Femto (Thermo Fisher Scientific, 34080 and 34095, respectively) on an ImageQuant RT ECL imaging system (GE Healthcare). Immunoblots were quantified by densitometry using ImageJ 1.50 (NIH). Optical density values for target proteins were normalized by actin and/or Ponceau staining as loading control. For each figure, all blots were processed in parallel and derive from the same experiment.

### Endoglycosidase-H sensibility assay

GCase N-glycan processing was conducted using an Endoglycosidase-H (Endo-H) sensitivity assay. Briefly, total homogenates from differentiated cells were digested with Endo-H enzyme for 2 h at 37 °C (P0702S; New England Biolabs). PNGase F (P0710S; New England Biolabs) was used as a control^8^. After digestion, GBA levels were analyzed by immunoblotting and percentage of EndoH-sensitive fraction was calculated.

### Cellular viability

Viability of differentiated cells was measured as the % of viable cells counting the number of viable and dead cells accumulated during the last 72 h prior to measurement and without changing the differentation media. Triplicates of at least three independent samples were analyzed in a Muse Cell Analyzer cytometer with the Muse Count & Viability Assay Kit (MCH100102, Thermofisher) that stains viable and non-viable cells based on their permeability to two DNA binding dyes present in the reagent.

### Immunofluorescence microscopy

Cells were fixed in 3% formaldehyde for 30 min at room temperature (RT). Following incubation with a blocking solution (powdered milk 0.2%, glycine 0.1 M, Triton X-100 0.01%, NCS 2%, and BSA 1%, in a 1× PBS solution) for 1 h at RT, the corresponding primary antibodies were used diluted in 0.1% BSA in 1× PBS overnight at 4 °C. Afterward, cells were incubated for 1 h at RT with the corresponding secondary antibodies, diluted in 0.1% BSA in 1× PBS. Thereafter, nuclei were labeled using Hoechst 33342 (62249; Thermo Fisher Scientific). Coverslips were then mounted using Dako Fluorescent Mounting Medium (S3023; Dako Denmark A/S, Glostrup, Denmark). Images were acquired using an Olympus FV1000 (Olympus Corporation, Tokyo, Japan) or a Zeiss LSM 980 confocal microscope (Carl Zeiss AG, Jena, Germany). Intensity and colocalization analyses were performed using ImageJ 1.50a (NIH, Bethesda, MD, USA) and Zen Blue (Carl Zeiss AG) software.

### In vivo cross-linking

In vivo cross-linking of synuclein oligomers was performed according to ref. ^75^ with disuccinimidyl glutarate (DSG).

### Filter retardation assay

Fifty micrograms of previously DSG-cross-linked cell extracts were subjected to a filter retardation assay following a previously described protocol^15^ using cellulose acetate membranes (0.2-μm pore size; GE Healthcare) in a Minifold-1 Dot-Blot System (Schleicher & Schuell GmbH, Keene, NH, USA). Next, membranes were washed twice with 1% SDS-PBS and immunodetection of different α-syn species was performed according to the protocol described above.

### Extracellular synuclein detection

Extracellular α -synuclein was detected from culture medium. After centrifugation to remove the remaining cells and debris, the medium was boiled and filtered in a nitrocellulose membrane using a Minifold-1 Dot-Blot System (Schleicher & Schuell GmbH). Finally, immunodetection was conducted following the standard immunoblot detection protocol.

### Cellular proteolytic assays

The degradation of long-lived proteins was quantified following a previously described protocol^76^. Radioactive pulse analysis was performed with [^3^H] valine (2 µ Ci/mL) (MT1654; HARTMANN ANALYTIC GmbH, Braunschweig, Germany) for 48 h at 37 °C. When indicated, cells were supplemented with 20 mM ammonium chloride and 100 μM leupeptin to block lysosomal-dependent proteolysis and 3-MA to inhibit macroautophagy. Lysosomal proteolysis was calculated as the proteolysis sensitive to inhibition by ammonium chloride and leupeptin, and CMA proteolysis as that which is sensitive to ammonium chloride and leupeptin and insensitive to 3-MA at the indicated time^77^.

### Lysosomal enzymatic activities

All enzymatic assays were conducted in triplicate in 96-well plates using 5–10 µg of total homogenate samples or 0.5–1 µg of the lysosomal-enriched fraction. Activity measurements for all enzymes were always corrected by mg of protein measured in parallel with the same sample by BCA.

#### Glucocerebrosidase activity (GCase):

Samples were resuspended in McIlvaine’s buffer (0.2 M citrate/phosphate buffer pH 5.4) containing 0.25% Triton X-100, 22 mM of the activator sodium taurocholate hydrate (SIGMA, 86339), and 5 mM of the fluorescent substrate 4-Methylumbelliferyl β-d-glucopyranoside (M363; Sigma-Aldrich). A sample volume of 100 μL per well was prepared and transferred to 96-well plates in triplicate and incubated for at least 1 h at 37 °C. Next, a stop solution (25 μL of 0.25 M glycine/NaOH pH 10.4) was added to terminate the reaction, and the liberated 4-methylumbelliferone was measured using an FLx800 spectrofluorimeter (BioTek, Winooski, VT, USA) at λex = 365 nm/λem = 450 nm. When indicated cells were treated 30 min with 1 mM conduritol β epoxide (CβE) (C5424; Sigma-Aldrich) before GCase assay.

#### Hexosaminidase activity assay:

Samples were dissolved in a reaction buffer containing 0.1 M acetate buffer pH 4.4, 1 mM of fluorescent substrate 4-methylumbelliferyl-N-acetyl-B-D-glucopyranoside (M2133; Sigma-Aldrich), and 0.125% Triton X-100. Stop solution (75 μL of 2 M glycine/Na_2_CO_3_) was added to terminate the reaction, and the plate was measured using an FLx800 spectrofluorimeter (BioTek) at λex = 365 nm, λem = 450 nm.

#### Acid phosphatase activity assay:

Samples were dissolved in citrate buffer solution 0.09 M pH 4.8 and substarte 4-nitrophenyl phosphate (1 tablet/2.5 mL buffer) (N9389; Sigma-Aldrich). Stop solution (200 μL 0.5 N NaOH) was added to terminate the reaction, and the plate was measured using an ELx800 spectrophotometer (BioTek) at λ = 405 nm.

#### Cathepsin B activity assay:

The activity was measured using a Cathepsin B Activity Assay Kit (ab65300; Abcam) according to the manufacturer’s instructions. Samples were processed without the cathepsin B inhibitor pepstatin A.

#### Acid sphingomyelinase activity assay:

Samples were dissolved in a reaction buffer containing 0.1 M NaAc, 0.2% sodium taurocholate hydrate (86339; Sigma-Aldrich; pH 5.2), and 110 μM of substrate HMU-PC (6-Hexadecanoylamino-4-methylumbelliferyl phosphorylcholine; Moscerdam Substrates, Rotterdam, Netherlands). Stop solution (200 μL glycine-NaOH 0.2 M, 0.2% m/v SDS, 0.2% v/v Triton X-100, pH 10.6) was added to terminate the reaction, the plate was measured using a SpectraMax M5 Multi-Mode Microplate Reader (Molecular Devices, San Jose, CA, USA) at λex = 355 nm, λem = 460 nm.

#### Acid ceramidase activity assay:

Samples were dissolved in a reaction buffer containing 0.1 M NaAc (pH 4.5) and 40 μM of substrate RBM14-C12 (860655; Avanti Polar Lipids Inc). Next, a stop solution (50 μL of methanol and 100 μL of a 2.5 mg/mL NaIO_4_ fresh solution in 100 mM glycine/NaOH buffer, pH 10.6) was added to terminate the reaction, after which the plate was measured using a SpectraMax M5 Multi-Mode Microplate Reader (Molecular Devices) at λex = 360 nm, λem = 446 nm.

#### Cathepsin D activity assay:

The activity was measured using a Cathepsin D Activity Assay Kit (ab65302; Abcam) according to the manufacturer’s instructions.

#### In situ Cathepsin D activity assay:

Cells were treated with a 1 μg/mL BODYPY-FL-pepstatin A probe (P12271; Thermo Fisher Scientific) for 1 h at 37 °C. Subsequently, flow cytometry analysis was performed using a BD LSR Fortessa Cell Analyzer (BD Biosciences); 10,000 cells were acquired, and fluorescence was determined using a 488-nm laser and 694/40 detector and represented as mean fluorescence intensity (MFI) using FCS Express software.

### Lysosomal pH measurement

Lysosomal pH was measured using LysoSensor Yellow/Blue DND-160 dye (L7545; Thermo Fisher Scientific) according to^76^.

### Quantitative real-time PCR (qPCR) analysis of LAMP-2A mRNA

Isolation of total messenger RNA (mRNA) was performed in cell pellets using the RNeasy Mini kit (Qiagen, Hilden, Germany), and reverse transcription was performed using 500 ng of total RNA and a High-Capacity cDNA Reverse Transcription kit (Thermo Fisher Scientific) according to manufacturer’s instructions. Real-time PCR was carried out using PowerUp SYBR Green Master Mix (Thermo Fisher Scientific) and a 7900HT Fast Real-Time PCR instrument (Applied Biosystems, Waltham, MA, USA) using LAMP-2A primers (Fw: GGGTTCAGCCTTTCAATGTG, Rev: CAGCATGATGGTGCTTGAGA), LAMP-2B primers (Fw: GGGTTCAGCCTTTCAATGTG and Rev: CCTGAAAGACCAGCACCAAC), and LAMP-2C primers (Fw: GTATTCTACAGCTGAAGAATGTTCTG and Rev: ACACCCACTGCAACAGGAAT). HPRT (Fw: TTATGGACAGGACTGAACGTCTTG, Rev: GCACACAGAGGGCTACAATGTG) and UBE2D2 (Fw: TGCCTGAGATTGCTCGGATCT, Rev: TCGCATACTTCTGAGTCCATTCC) were used as reference genes. Quantification was performed using the comparative CT method. All the samples were tested in triplicate and the relative expression values were normalized to the expression value of HPRT and UBE2D2. The fold change was calculated using the 2^-ΔCt^ method. Three independent experiments with differentiated cells were performed.

### Cholesterol quantification

Cholesterol levels were measured using an Amplex Red Cholesterol Assay Kit (A12216; Thermo Fisher Scientific) according to the manufacturer’s instructions.

### Transfection

Plasmid expressing human LAMP-2 cDNA (transcript variant A; SC118738; OriGene, Rockville, MD, USA) was transiently transfected into BE(2)-M17 cell lines using Lipofectamine 3000 Reagent (L3000015; Thermo Fisher Scientific) according to the manufacturer’s protocol.

### Statistical analysis

All values are expressed as the mean ± standard error of the mean (SEM). Significant differences (*p < 0.05, **p < 0.01, ***p < 0.001, ****p < 0.0001) when comparing two groups were determined by a two-tailed unpaired Student’s *t*-test. When comparing more than two groups, significant differences were determined by one- or two-way ANOVA followed by Tukey’s multiple comparisons test. Statistical analyses were performed using GraphPad Prism 6 (GraphPad Software Inc, San Diego, CA, USA).

## Supplementary information

SUPPLEMENTAL MATERIAL

## Data Availability

The data that support the findings of this study does not present restrictions and are available upon a request for information that is reasonably made and that can be provided by the corresponding author.

## References

[1] Tysnes OB, Storstein A (2017). Epidemiology of Parkinson’s disease. J. Neural Transm..

[2] Dehay B (2015). Targeting α-synuclein for treatment of Parkinson’s disease: mechanistic and therapeutic considerations. Lancet Neurol..

[3] Neumann J (2009). Glucocerebrosidase mutations in clinical and pathologically proven Parkinson’s disease. Brain.

[4] Sidransky E (2009). Multicenter analysis of glucocerebrosidase mutations in Parkinson’s disease. N. Engl. J. Med..

[5] Lesage S (2011). Large-scale screening of the Gaucher’s disease-related glucocerebrosidase gene in Europeans with Parkinson’s disease. Hum. Mol. Genet..

[6] Stojkovska I, Krainc D, Mazzulli JR (2018). Molecular mechanisms of α-synuclein and GBA1 in Parkinson’s disease. Cell Tissue Res..

[7] Bae E-J (2014). Glucocerebrosidase depletion enhances cell-to-cell transmission of α-synuclein. Nat. Commun..

[8] Ron I, Horowitz M (2005). ER retention and degradation as the molecular basis underlying Gaucher disease heterogeneity. Hum. Mol. Genet..

[9] Cullen V (2011). Acid β-glucosidase mutants linked to Gaucher disease, Parkinson disease, and Lewy body dementia alter α-synuclein processing. Ann. Neurol..

[10] Gegg ME, Menozzi E, Schapira AHV (2022). Glucocerebrosidase-associated Parkinson disease: Pathogenic mechanisms and potential drug treatments. Neurobiol. Dis..

[11] Körschen HG (2013). The non-lysosomal β-glucosidase GBA2 is a non-integral membrane-associated protein at the endoplasmic reticulum (ER) and Golgi. J. Biol. Chem..

[12] Maley F, Trimble RB, Tarentino AL, Plummer TH (1989). Characterization of glycoproteins and their associated oligosaccharides through the use of endoglycosidases. Anal. Biochem..

[13] Migdalska-Richards, A. & Schapira, A. H. V. V. The relationship between glucocerebrosidase mutations and Parkinson disease. *J. Neurochem.* 77–90 10.1111/jnc.13385 (2016). 10.1111/jnc.13385 PMC5111601 26860875

[14] Hetz C, Zhang K, Kaufman RJ (2020). Mechanisms, regulation and functions of the unfolded protein response. Nat. Rev. Mol. Cell Biol..

[15] Recasens A (2018). Lack of pathogenic potential of peripheral α-synuclein aggregates from Parkinson’s disease patients. Acta Neuropathol. Commun..

[16] Navarro-Romero A, Montpeyó M, Martinez-Vicente M (2020). The emerging role of the lysosome in Parkinson’s Disease. Cells.

[17] Martinez-Vicente M, Vila M (2013). Alpha-synuclein and protein degradation pathways in Parkinson’s disease: a pathological feed-back loop. Exp. Neurol..

[18] Auteri JS, Okada A, Bochaki V, Dice JF (1983). Regulation of intracellular protein degradation in IMR-90 human diploid fibroblasts. J. Cell Physiol..

[19] Aits S (2015). Sensitive detection of lysosomal membrane permeabilization by lysosomal galectin puncta assay by lysosomal galectin puncta assay. Autophagy.

[20] Fernandes HJRR (2016). ER stress and autophagic perturbations lead to elevated extracellular α-Synuclein in GBA-N370S Parkinson’s iPSC-derived dopamine neurons. Stem Cell Rep..

[21] Magalhaes J (2016). Autophagic lysosome reformation dysfunction in glucocerebrosidase deficient cells: relevance to Parkinson disease. Hum. Mol. Genet..

[22] Schöndorf DC (2014). IPSC-derived neurons from GBA1-associated Parkinson's disease patients show autophagic defects and impaired calcium homeostasis. Nat. Commun.

[23] Cuervo AM, Stefanis L, Fredenburg R, Lansbury PT, Sulzer D (2004). Impaired degradation of mutant alpha-synuclein by chaperone-mediated autophagy. Science.

[24] Vogiatzi T, Xilouri M, Vekrellis K, Stefanis L (2008). Wild type alpha-synuclein is degraded by chaperone-mediated autophagy and macroautophagy in neuronal cells. J. Biol. Chem..

[25] Mak SK, McCormack AL, Manning-Bog AB, Cuervo AM, Di Monte DA (2010). Lysosomal degradation of alpha-synuclein in vivo. J. Biol. Chem..

[26] Kaushik S (2011). Chaperone-mediated autophagy at a glance. J. Cell Sci..

[27] Patel B, Cuervo AM (2015). Methods to study chaperone-mediated autophagy. Methods.

[28] Rodriguez-Navarro JA, Cuervo AM (2012). Dietary lipids and aging compromise chaperone-mediated autophagy by similar mechanisms. Autophagy.

[29] Kaushik S, Massey AC, Cuervo AM (2006). Lysosome membrane lipid microdomains: novel regulators of chaperone-mediated autophagy. EMBO J..

[30] Anguiano J (2013). Chemical modulation of chaperone-mediated autophagy by retinoic acid derivatives. Nat. Chem. Biol..

[31] Gong Z (2018). Humanin is an endogenous activator of chaperonemediated autophagy. J. Cell Biol..

[32] Filograna R (2015). Analysis of the catecholaminergic phenotype in human SH-SY5Y and BE(2)-M17 neuroblastoma cell lines upon differentiation. PLoS ONE.

[33] Muñoz, S. S., Petersen, D., Marlet, F. R., Kücükköse, E. & Galvagnion, C. The interplay between Glucocerebrosidase, α-synuclein and lipids in human models of Parkinson’s disease. *Biophys. Chem.*10.1016/j.bpc.2020.106534 (2021). 10.1016/j.bpc.2020.106534 33832803

[34] Enquist IB (2007). Murine models of acute neuronopathic Gaucher disease. Proc. Natl Acad. Sci. USA.

[35] Farfel-Becker T (2014). Neuronal accumulation of glucosylceramide in a mouse model of neuronopathic Gaucher disease leads to neurodegeneration. Hum. Mol. Genet..

[36] Sun Y (2012). Ex vivo and in vivo effects of isofagomine on acid β-glucosidase variants and substrate levels in Gaucher disease. J. Biol. Chem..

[37] Sun, Y. et al. Substrate compositional variation with tissue/region and Gba1 mutations in mouse models-implications for Gaucher disease. *PLoS ONE.*10.1371/journal.pone.0057560 (2013). 10.1371/journal.pone.0057560 PMC3592923 23520473

[38] Polinskii NK (2021). Decreased glucocerebrosidase activity and substrate accumulation of glycosphingolipids in a novel gba1 d409v knock-in mouse model. PLoS ONE.

[39] Sardi SP (2011). CNS expression of glucocerebrosidase corrects alpha-synuclein pathology and memory in a mouse model of Gaucher-related synucleinopathy. Proc. Natl Acad. Sci. USA.

[40] Nilsson O, Svennerholm L (1982). Accumulation of glucosylceramide and glucosylsphingosine (psychosine) in cerebrum and cerebellum in infantile and juvenile Gaucher disease. J. Neurochem..

[41] Orvisky E (2002). Glucosylsphingosine accumulation in tissues from patients with Gaucher disease: correlation with phenotype and genotype. Mol. Genet. Metab..

[42] Huebecker M (2019). Reduced sphingolipid hydrolase activities, substrate accumulation and ganglioside decline in Parkinson’s disease. Mol. Neurodegener..

[43] Rocha EM (2015). Progressive decline of glucocerebrosidase in aging and Parkinson’s disease. Ann. Clin. Transl. Neurol..

[44] Gegg ME (2015). No evidence for substrate accumulation in Parkinson brains with GBA mutations. Mov. Disord..

[45] Kurzawa-Akanbi M (2021). Altered ceramide metabolism is a feature in the extracellular vesicle-mediated spread of alpha-synuclein in Lewy body disorders. Acta Neuropathol..

[46] Clark, L. N. et al. Gene-wise association of variants in four lysosomal storage disorder genes in neuropathologically confirmed Lewy body disease. *PLoS ONE.*10.1371/journal.pone.0125204 (2015). 10.1371/journal.pone.0125204 PMC4416714 25933391

[47] Lloyd-Evans E, Pelled D, Riebeling C, Futerman AH (2003). Lyso-glycosphingolipids mobilize calcium from brain microsomes via multiple mechanisms. Biochem. J.

[48] Korkotian, E. et al. Elevation of intracellular glucosylceramide levels results in an increase in endoplasmic reticulum density and in functional calcium stores in cultured neurons. *J. Biol. Chem.*10.1074/jbc.274.31.21673 (1999). 10.1074/jbc.274.31.21673 10419477

[49] Pelled D (2005). Enhanced calcium release in the acute neuronopathic form of Gaucher disease. Neurobiol. Dis..

[50] Hannun YA, Obeid LM (2018). Sphingolipids and their metabolism in physiology and disease. Nat. Rev. Mol. Cell Biol..

[51] Esmaeili, Y. et al. Targeting autophagy, oxidative stress, and ER stress for neurodegenerative diseases treatment. *J. Control. Release.*10.1016/j.jconrel.2022.03.001 (2022). 10.1016/j.jconrel.2022.03.001 35248646

[52] Kuo SH (2022). Mutant glucocerebrosidase impairs α-synuclein degradation by blockade of chaperone-mediated autophagy. Sci. Adv.

[53] García-Sanz P, Orgaz L, Bueno-gil G, Espadas I (2017). N370S-GBA1 mutation causes lysosomal cholesterol accumulation in Parkinson’s disease. Mov. Disord.

[54] Awad, O. et al. Altered TFEB-mediated lysosomal biogenesis in Gaucher disease iPSC-derived neuronal cells. *Hum. Mol. Genet.*10.1093/hmg/ddv297 (2015). 10.1093/hmg/ddv297 26220978

[55] Mazzulli JR (2011). Gaucher disease glucocerebrosidase and α-synuclein form a bidirectional pathogenic loop in synucleinopathies. Cell.

[56] Shibata M (2006). Regulation of intracellular accumulation of mutant Huntingtin by Beclin 1. J. Biol. Chem..

[57] Yang, S., Gegg, M., Chau, D. & Schapira, A. Glucocerebrosidase activity, cathepsin D and monomeric α- synuclein interactions in a stem cell derived neuronal model of a PD associated GBA1 mutation. *Neurobiol. Dis.* 104620. 10.1016/j.nbd.2019.104620 (2019). 10.1016/j.nbd.2019.104620 PMC6983928 31634558

[58] Wolfe DM (2013). Autophagy failure in Alzheimer’s disease and the role of defective lysosomal acidification. Eur. J. Neurosci..

[59] Dehay B (2012). Lysosomal dysfunction in Parkinson disease: ATP13A2 gets into the groove. Autophagy.

[60] Li H (2019). Mitochondrial dysfunction and mitophagy defect triggered by heterozygous GBA mutations. Autophagy.

[61] Kim MJ, Jeon S, Burbulla LF, Krainc D (2018). Acid ceramidase inhibition ameliorates a -synuclein accumulation upon loss of GBA1 function. Hum. Mol. Genet..

[62] Massey AC, Kaushik S, Sovak G, Kiffin R, Cuervo AM (2006). Consequences of the selective blockage of chaperone-mediated autophagy. Proc. Natl Acad. Sci. USA.

[63] Mak SK, McCormack AL, Manning-Bog AB, Cuervo AM, Di Monte DA (2010). Lysosomal degradation of α-synuclein in vivo. J. Biol. Chem..

[64] Martinez-Vicente M (2008). Dopamine-modified α-synuclein blocks chaperone-mediated autophagy. J. Clin. Investig..

[65] Xilouri M (2016). Impairment of chaperone-mediated autophagy induces dopaminergic neurodegeneration in rats. Autophagy.

[66] Orenstein SJ (2013). Interplay of LRRK2 with chaperone-mediated autophagy. Nat. Neurosci..

[67] Kabuta T, Furuta A, Aoki S, Furuta K, Wada K (2008). Aberrant interaction between Parkinson disease-associated mutant UCH-L1 and the lysosomal receptor for chaperone-mediated autophagy. J. Biol. Chem..

[68] Cuervo AM, Stafanis L, Fredenburg R, Lansbury PT, Sulzer D (2004). Impaired degradation of mutant α-synuclein by chaperone-mediated autophagy. Science.

[69] Rodriguez-Navarro JA (2012). Inhibitory effect of dietary lipids on chaperone-mediated autophagy. Proc. Natl Acad. Sci. USA.

[70] Klionsky, D. J. et al. Guidelines for the use and interpretation of assays for monitoring autophagy (4th edition). *Autophagy.*10.1080/15548627.2020.1797280 (2021). 10.1080/15548627.2020.1797280 PMC7996087 33634751

[71] Man, W. K. et al. A role of cholesterol in modulating the binding of α-synuclein to synaptic-like vesicles. *Front. Neurosci.*10.3389/fnins.2020.00018 (2020). 10.3389/fnins.2020.00018 PMC7000551 32063829

[72] Jakubec, M. et al. Cholesterol-containing lipid nanodiscs promote an α-synuclein binding mode that accelerates oligomerization. *FEBS J.*10.1111/febs.15551 (2020). 10.1111/febs.15551 32892498

[73] Gorrochategui E, Casas J, Porte C, Lacorte S, Tauler R (2015). Chemometric strategy for untargeted lipidomics: Biomarker detection and identification in stressed human placental cells. Anal. Chim. Acta.

[74] Storrie, B. & Madden, E. A. Isolation of subcellular organelles. *Methods Enzymol.*10.1016/0076-6879(90)82018-W (1990). 10.1016/0076-6879(90)82018-w 2156127

[75] Dettmer U, Newman AJ, Luth ES, Bartels T, Selkoe D (2013). In vivo cross-linking reveals principally oligomeric forms of α-synuclein and β-synuclein in neurons and non-neural cells. J. Biol. Chem..

[76] Dehay B (2012). Loss of P-type ATPase ATP13A2/PARK9 function induces general lysosomal deficiency and leads to Parkinson disease neurodegeneration. Proc. Natl Acad. Sci. USA.

[77] Kaushik, S. & Cuervo, A. M. Methods to monitor chaperone-mediated autophagy. In *Methods in Enzymology.* Vol. 452 (Elsevier Inc., 2009). 10.1016/S0076-6879(08)03619-7 PMC4300957 19200890

