# Supplementary material for: Lysosomal lipid alterations caused by glucocerebrosidase deficiency promote lysosomal dysfunction, chaperone-mediated-autophagy deficiency, and alpha-synuclein pathology
Source: NPJ Parkinsons Dis. 2022 Oct 6;8:126. doi: 10.1038/s41531-022-00397-6 (PMC9537323; doi:10.1038/s41531-022-00397-6)

Supplementary Figures:

**Figure S1**	**Validation of GBA1-specific GCase activity and lysosomal enriched fractions**
**Figure S2**	**Loss of GBA activity leads to cathepsin B and D deficiency.**
**Figure S3**	**Expression of LAMP-2A is not affected in differentiated GBA deficient cells**
**Figure S4**	**Graphical Abstract.**

Supplementary Information: full, uncropped main western blots

		**Panel**
**Figure 1**	Loss of lysosomal GCase activity leads to substrate accumulation in GBA mutant cells	Figure 1a
**Figure 2**	Mutant GBA proteins are retained in the ER	Figure 2a
		Figure 2c
**Figure 3**	Mutant GBA accumulates different α-syn species	Figure 3a
		Figure 3c
**Figure 5**	Mutant GBA causes alterations in macroautophagy	Figure 5a
		Figure 5b
**Figure 6**	Mutant GBA is associated with CMA impairment	Figure 6a
		Figure 6e
		Figure 6h
**Figure S1b**	Validation of GBA1-specific GCase activity and lysosomal enriched fractions	Figure S1b
**Figure S2b**	Loss of GBA activity leads to cathepsin B and D deficiency	Figure S2b

Supplementary Figure 1

A

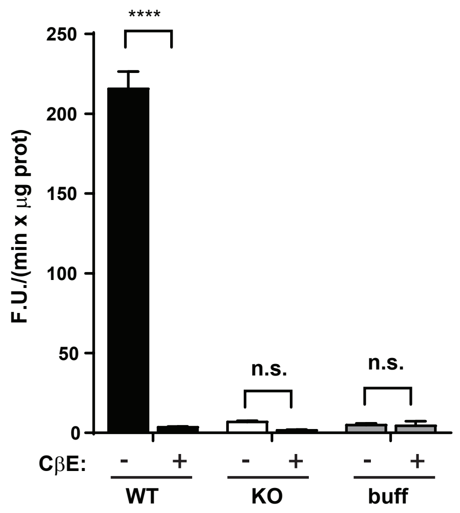

B

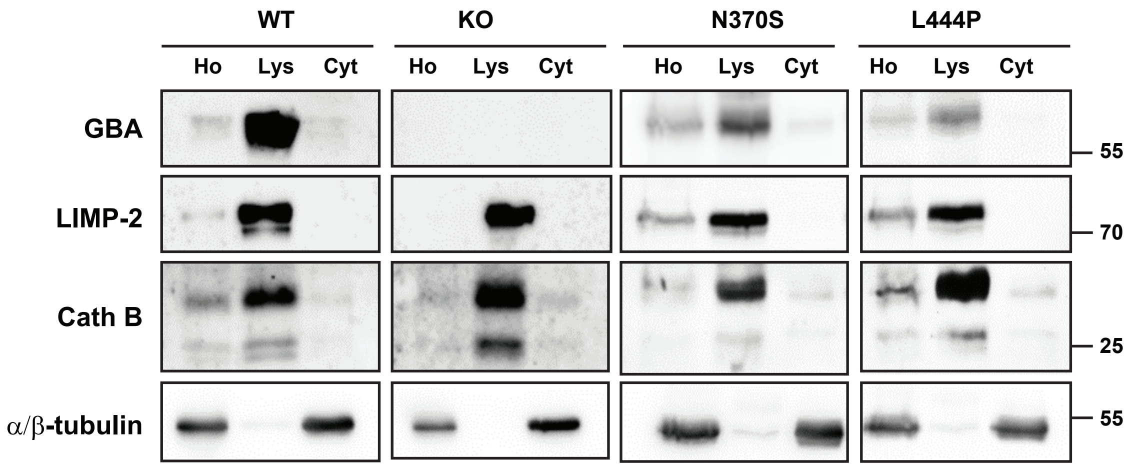

Supplementary Figure 1: Validation of GBA1-specific GCase activity and lysosomal enriched fractions.
(A) GCase activity in the presence of GBA1-specific inhibitor Conduritol β epoxide (CβE): GCase activity in the presence and absence of 1 mM CβE inhibitor (30 min) in GBA-WT total homogenate, GBA-KO (only without expression of *GBA1* gene) total homogenate and control sample without cellular homogenate but with GCase buffer and substrate. Activity is expressed as fluorescence unit (F.U.) per minut and total protein μg. n = 3 in each group. Data is presented as mean ± s.e.m. **** $p < 0.0001$ after one-way ANOVA followed by Tukey's multiple comparisons test.
(B) validation of isolated of lysosomal fractions: immunodetection of GBA protein, two lysosomal markers (LIMP-2 and mature isoforms of cathepsin B), and cytosolic marker (α/β-tubulin) in total homogenate (Ho) and two subcellular fractions: lysosomal enriched fraction (Lys) and cytosol fraction (Cyt) isolated from all cell lines.

Supplementary Figure 2

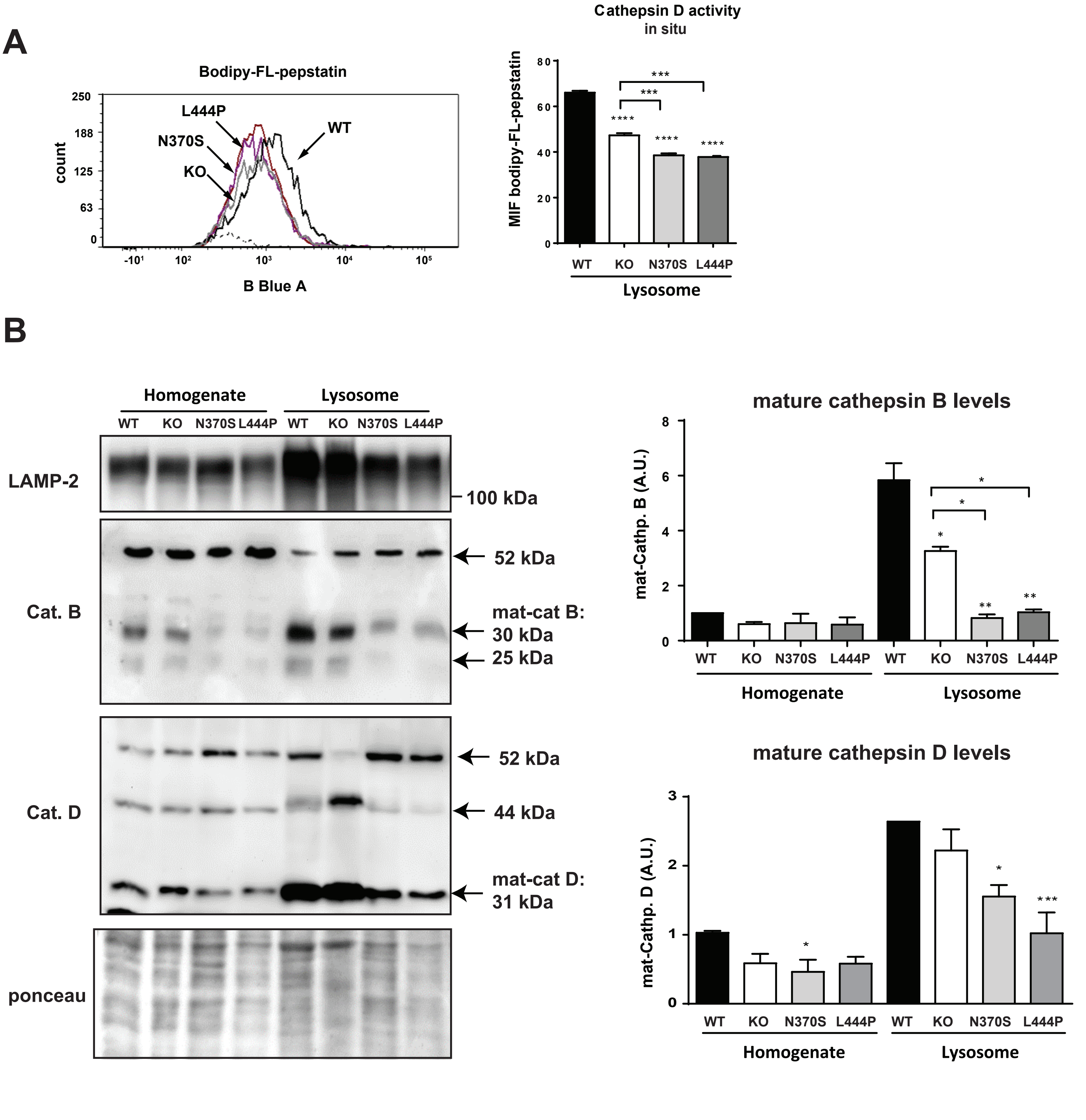

Supplemental Figure S2: Loss of GBA activity leads to cathepsin B and D deficiency.
(A) Cathepsin D activity assay in situ using BODYPY-FL-pepstatin A substrate determined by flow cytometry. (B) Cathepsin B and D protein levels determined by immunodetection in total homogenate and isolated lysosomal fractions. Mature cathepsin forms are indicated (mat-cat B and mat-cat D) and quantified (right).

Supplementary Figure 3

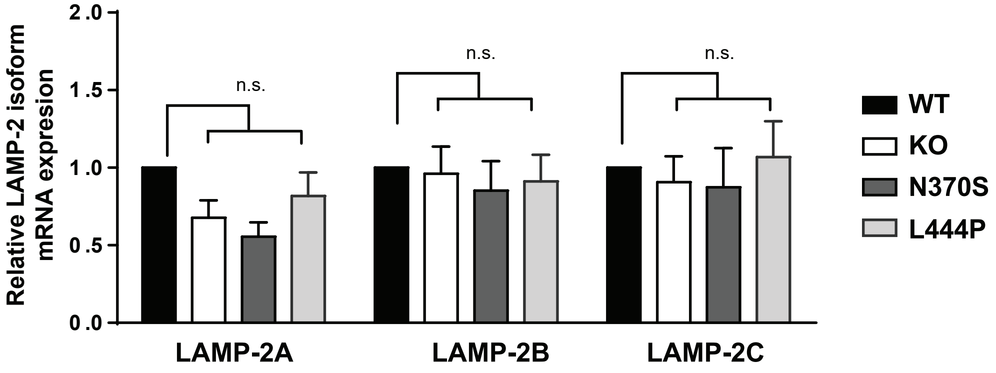

Supplementary Figure 3: Expression of LAMP-2A is not affected in differentiated GBA deficient cells. Determination of human LAMP-2 gene expression comprising the transcript variants A, B, and C by real-time reverse transcription-polymerase chain reaction (qPCR). Data are presented as mean ± s.e.m values of at least three independent experiments, and two-way ANOVA followed by Tukey's multiple comparisons test was conducted. No statistically significant differences were observed between the different groups.

Supplementary Figure 4:

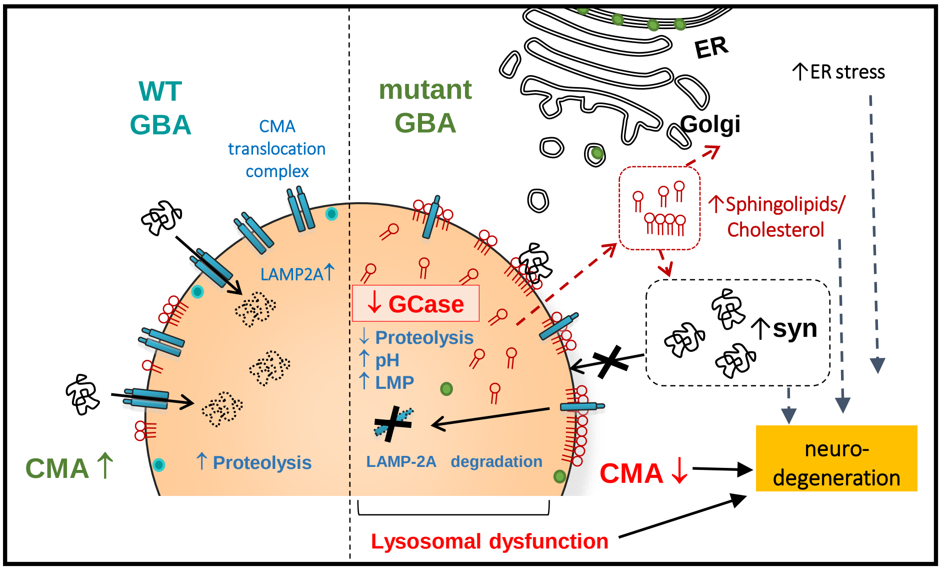

Supplementary Figure 4: Graphical Abstract. The loss of GCase activity generates extensive lysosomal dysfunction and an increase in the levels of sphingolipids and cholesterol in the lysosomal membrane that directly affects the normal dynamics of the CMA receptor LAMP-2A, impairing CMA activity and consequently synuclein lysosomal-dependent degradation. This is a new molecular mechanisms which is added to other converging events, where GCase activity loss promotes α-syn accumulation, contributing to neurodegeneration in Parkinson.

Supplementary Information: full, uncropped main western blots

Figure 1a:

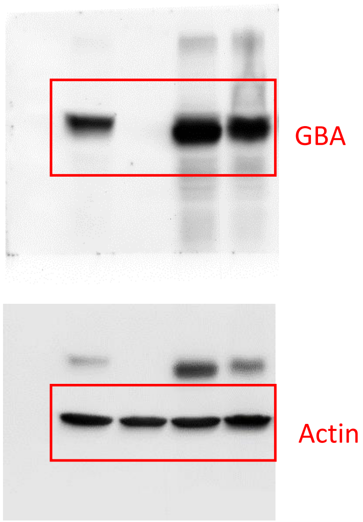

Figure 2a:

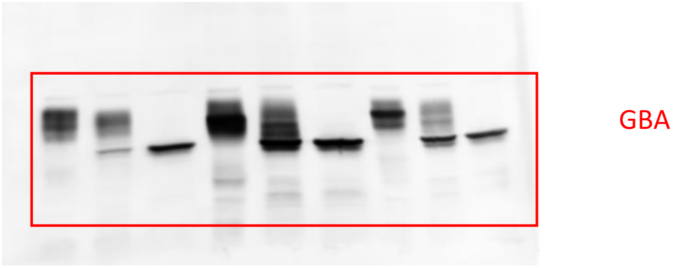

Figure 2c:

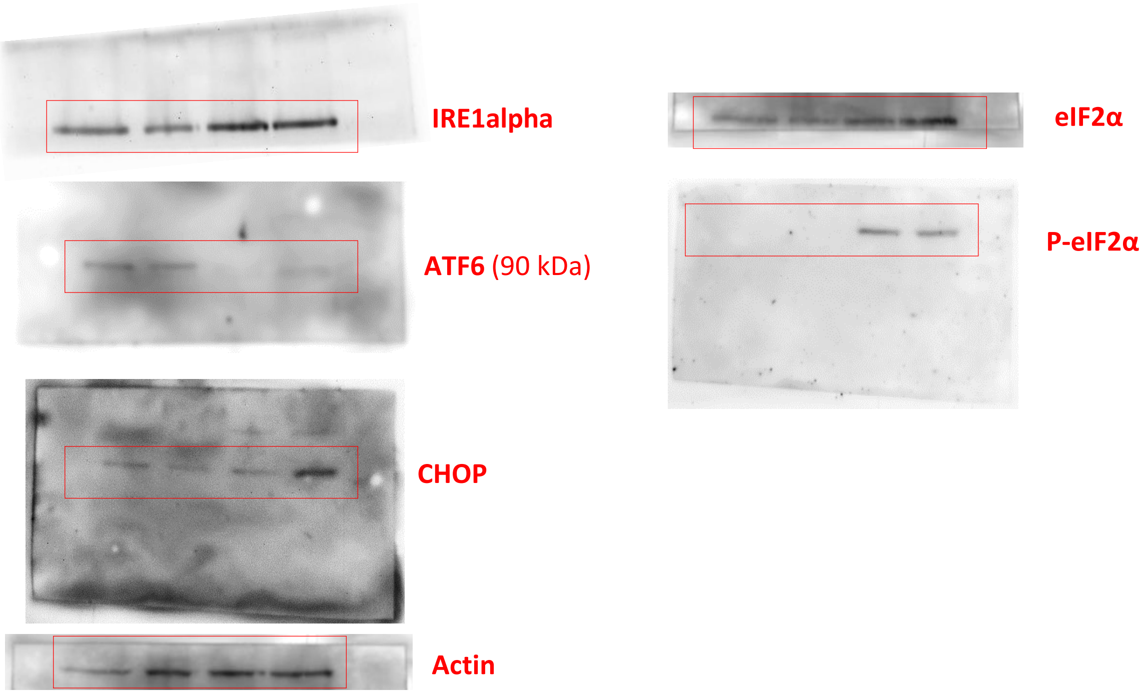

Figure 3a:

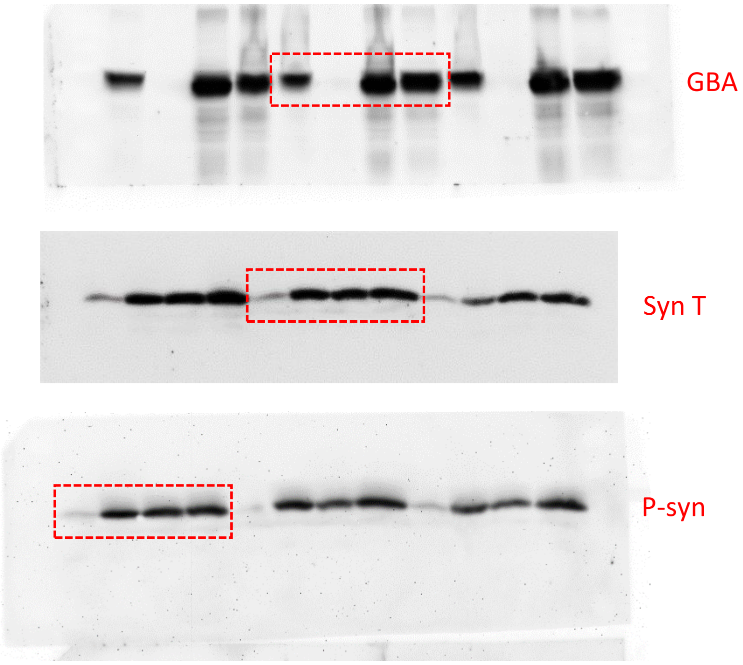

Figure 3c:

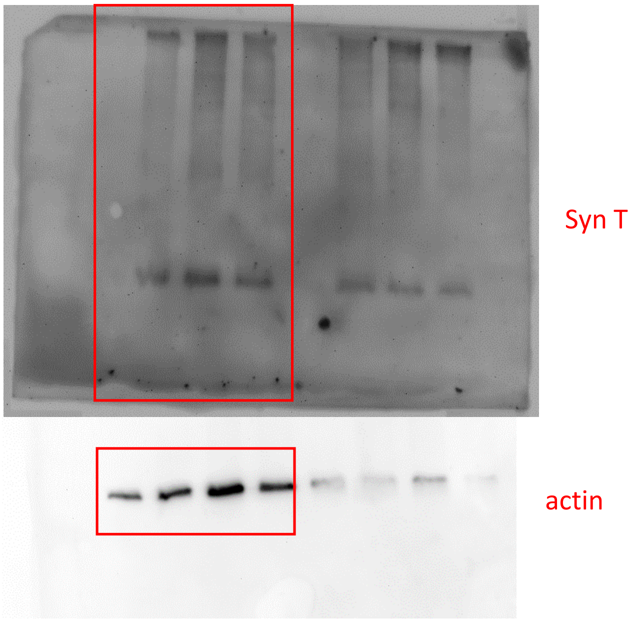

Figure 5a:

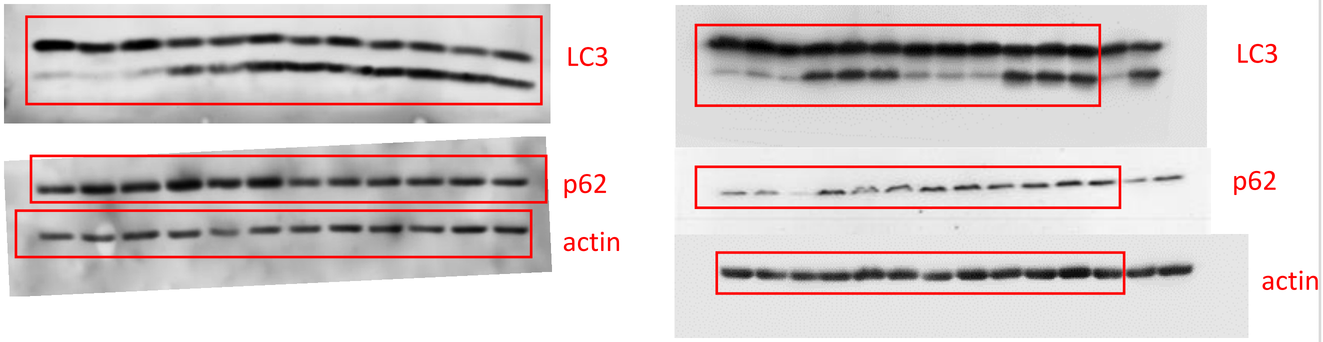

Figure 5b:

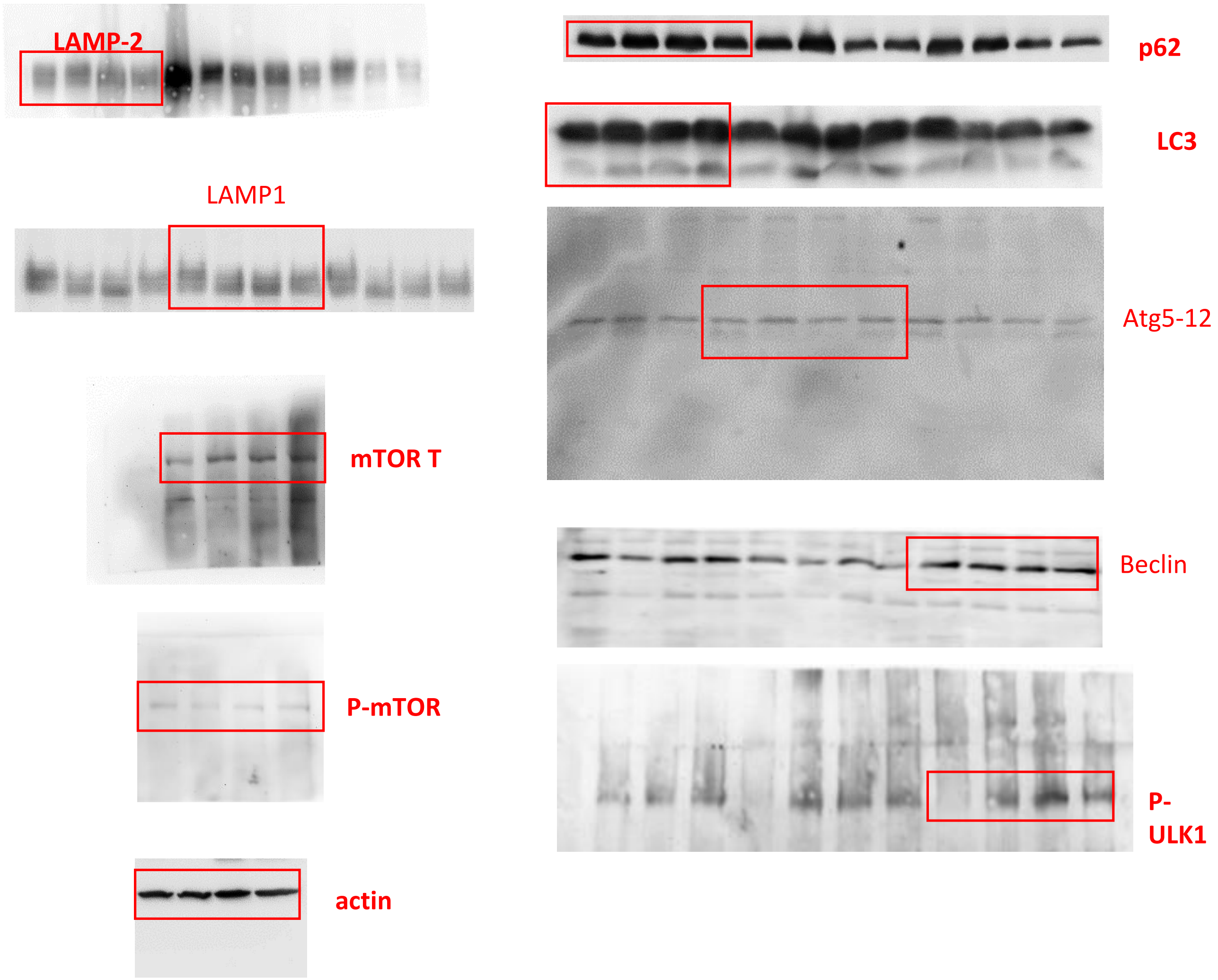

Figure 6a:

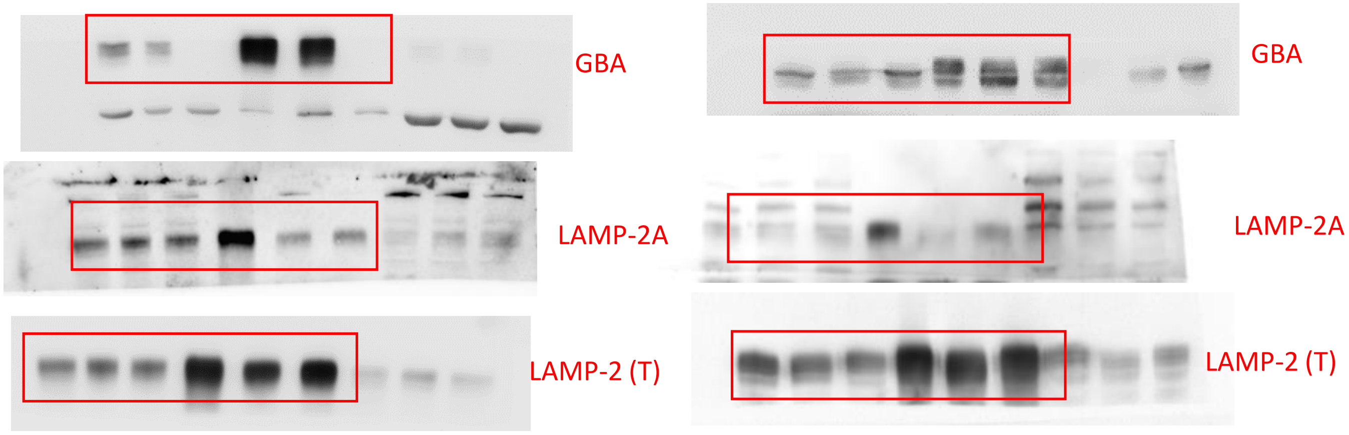

Figure 6e:

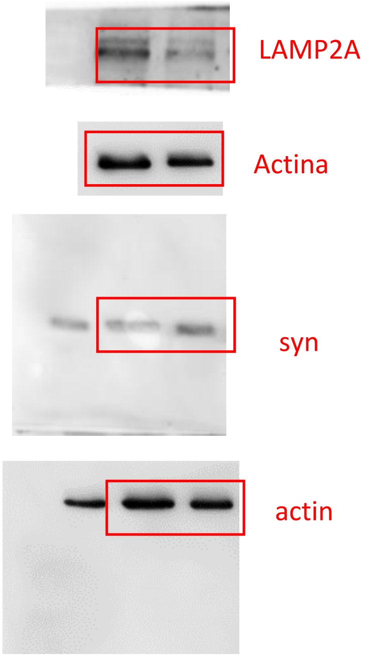

Figure 6g:

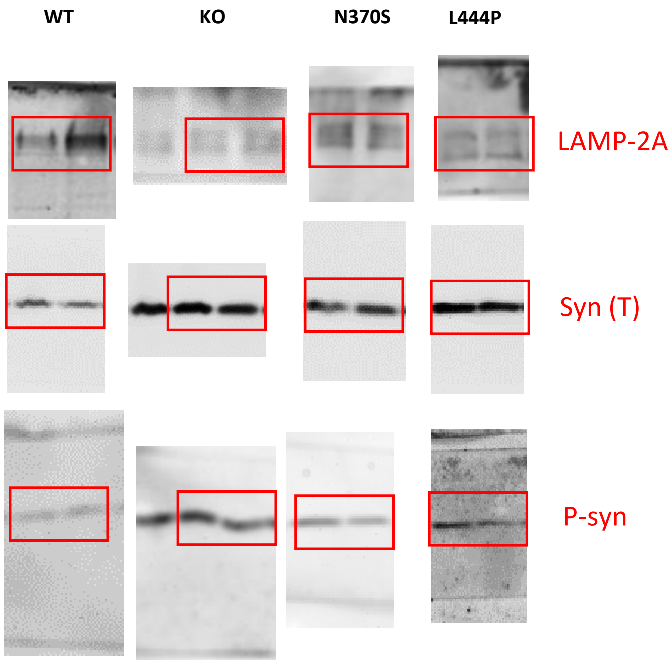

Figure 6h:

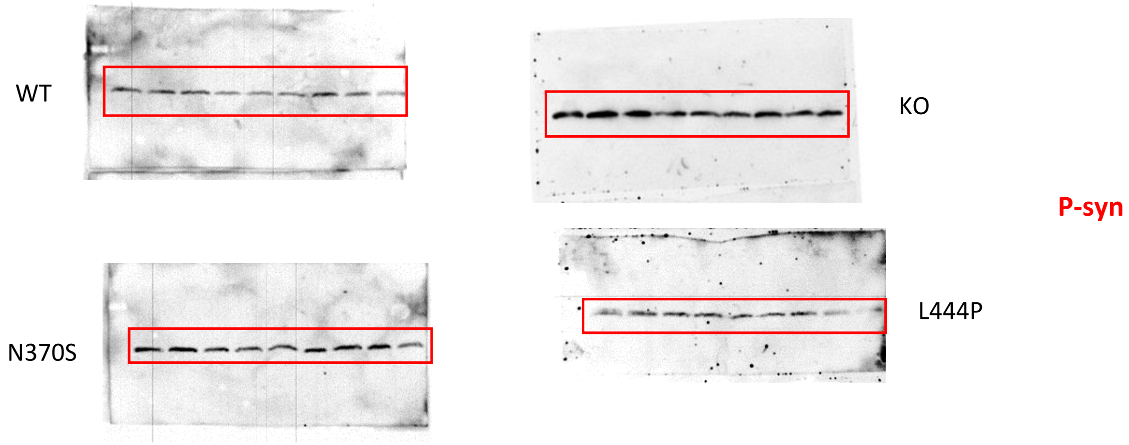

Figure S1a:

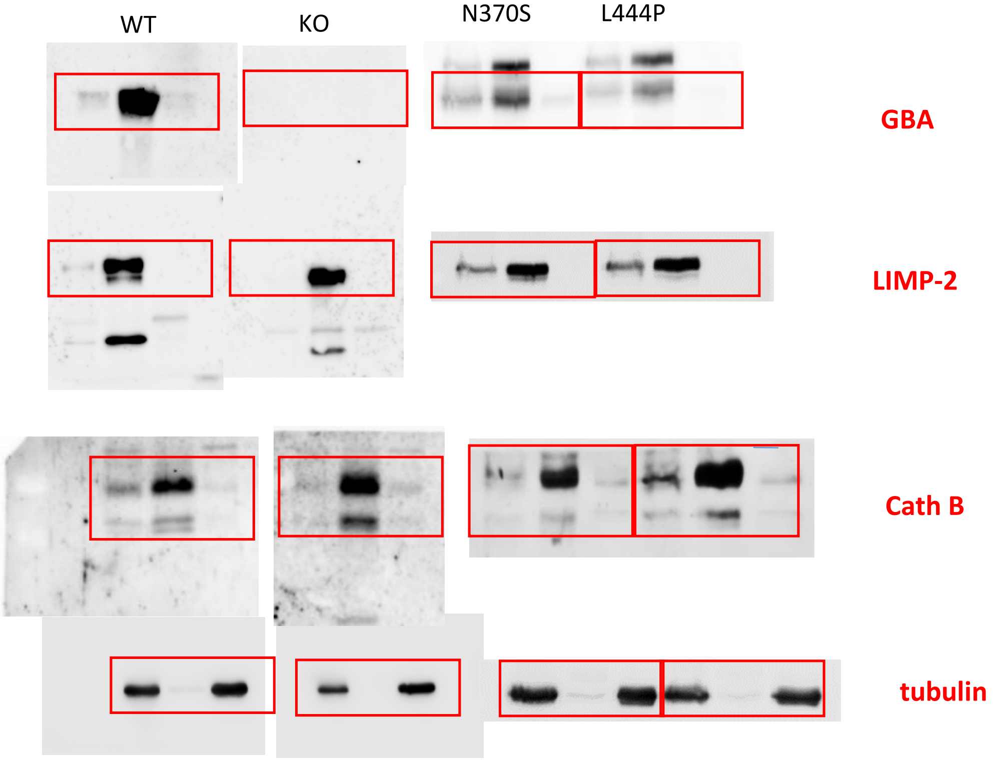

Figure S2b:

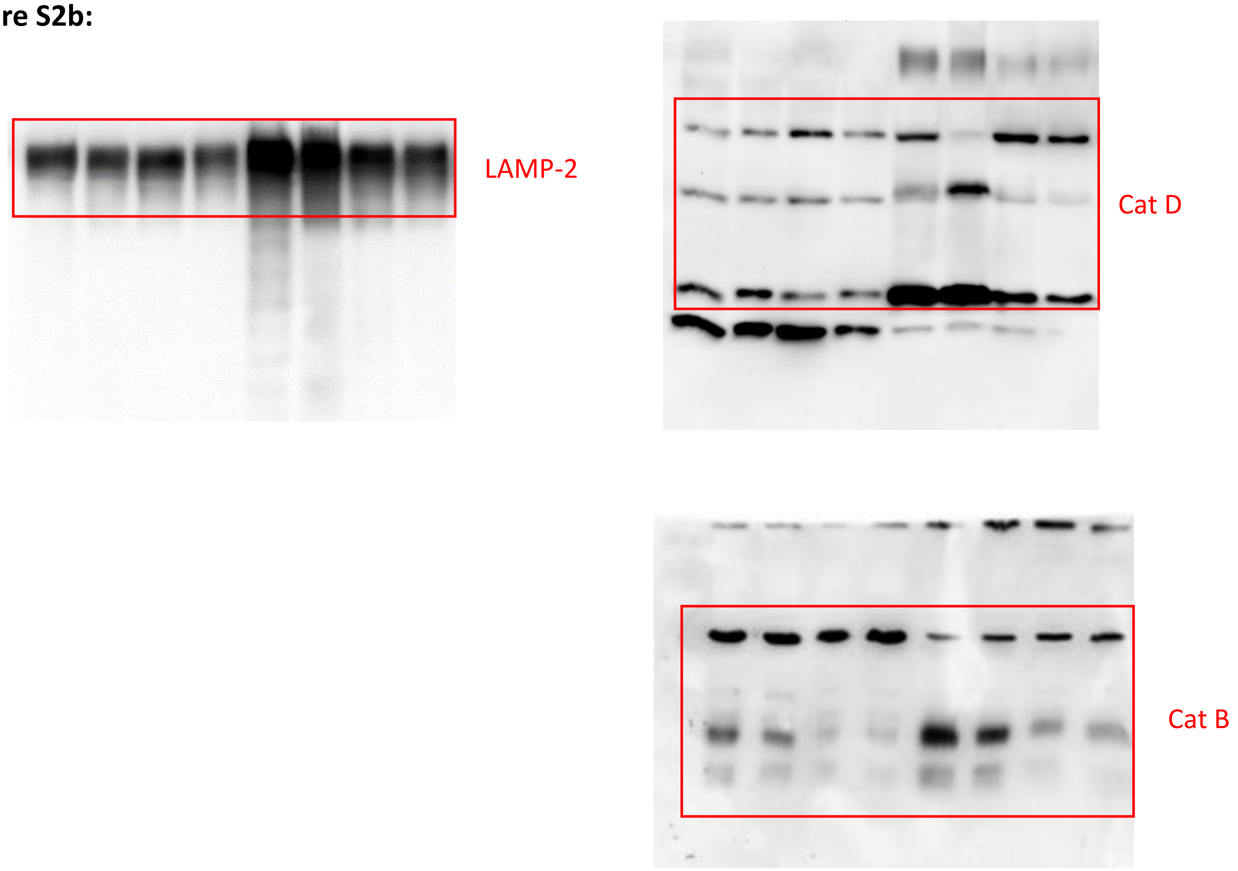

Supplement: Supplementary file 1 — SUPPLEMENTAL MATERIAL [file 41531_2022_397_MOESM1_ESM.pdf]
